# Reovirus infection of tumor cells reduces the expression of NKG2D ligands, leading to impaired NK-cell cytotoxicity and functionality

**DOI:** 10.3389/fimmu.2023.1231782

**Published:** 2023-09-11

**Authors:** Raghad Khaleafi, Jelena Zeleznjak, Sapir Cordela, Shani Drucker, Tihana Lenac Rovis, Stipan Jonjic, Yotam Bar-On

**Affiliations:** ^1^ Department of Immunology, Rappaport Faculty of Medicine, Technion-Israel Institute of Technology, Haifa, Israel; ^2^ Department of Histology and Embryology/Center for Proteomics, Faculty of Medicine, University of Rijeka, Rijeka, Croatia

**Keywords:** oncolytic viruses, innate immunity, NK cells, immune evasion, reovirus, tumor microenvironment

## Abstract

In recent years, reoviruses have been of major interest in immunotherapy because of their oncolytic properties. Preclinical and clinical trials, in which reovirus was used for the treatment of melanoma and glioblastoma, have paved the way for future clinical use of reovirus. However, little is known about how reovirus infection affects the tumor microenvironment and immune response towards infected tumor cells. Studies have shown that reovirus can directly stimulate natural killer (NK) cells, but how reovirus affects cellular ligands on tumor cells, which are ultimately key to tumor recognition and elimination by NK cells, has not been investigated. We tested how reovirus infection affects the binding of the NK Group-2 member D (NKG2D) receptor, which is a dominant mediator of NK cell anti-tumor activity. Using models of human-derived melanoma and glioblastoma tumors, we demonstrated that NKG2D ligands are downregulated in tumor cells post-reovirus-infection due to the impaired translation of these ligands in reovirus-infected cells. Moreover, we showed that downregulation of NKG2D ligands significantly impaired the binding of NKG2D to infected tumor cells. We further demonstrated that reduced recognition of NKG2D ligands significantly alters NK cell anti-tumor cytotoxicity in human primary NK cells and in the NK cell line NK-92. Thus, this study provides novel insights into reovirus-host interactions and could lead to the development of novel reovirus-based therapeutics that enhance the anti-tumor immune response.

## Introduction

Mammalian orthoreovirus, henceforth reovirus, is a member of the Reoviridae family of viruses. Reovirus is usually constricted to the respiratory and enteric pathways but is generally considered nonpathogenic. It is composed of two concentric protein capsids and a genome consisting of ten strands of double-stranded RNA (1–3). Upon infection of mammalian cells, viral RNA triggers an anti-viral response in host cells, marked by the initiation of type I and type III IFN responses, which were shown to halt the replication of reovirus in enterocytes (1). Another effect triggered by the double-stranded RNA structures is the activation of protein kinase RNA-activated (PKR), which phosphorylates the cellular translation initiation factor eIF2α. Phosphorylated eIF2α inhibits translation in the cell, thus preventing the virus from producing viral proteins and ultimately stopping the propagation of new viral units (4). However, RAS oncogenes block PKR activity, thus preventing the translational shutdown observed after reovirus infection (5). Therefore, reovirus has been shown to replicate with higher efficiency in cells harboring an activated RAS pathway, including many cancers and cancer cell lines that are preferentially infected with the reovirus (6). As the RAS pathway promotes proliferation, its activation remains a critical event in cancer transformation (7). Mutations in RAS genes are present in 19% of all tumors (8). 15-25% of melanoma tumors possess an activating mutation in the NRAS gene (9); 45-50% of colorectal tumors possess activating mutation KRAS (10), and in certain tumors, such as glioblastoma, the RAS pathway can be highly activated even without a direct mutation in the RAS genes (7).

This, along with the fact that reovirus type-3 Dearing (T3D) is a naturally lytic virus, has led to the prospect of using reoviruses as anti-tumor agents (11). A seminal study by Coffey et al. demonstrated that a single intratumoral injection of the reovirus could result in regression of human U87-MG glioblastoma tumors in immunodeficient (SCID) mice (12). Similarly, they further demonstrated that multiple reovirus injections in immunocompetent mice could lead to complete tumor regression (13). Other preclinical studies have further supported the therapeutic potential of reovirus-based therapy against melanoma, glioblastoma, breast cancer, head and neck cancer, prostate cancer, and other types of cancer (12, 14–17), but have also revealed several limitations to the use of reovirus as a single therapeutic agent (13, 16–18). Notably, in the last 20 years, a clinical-grade formulation of the T3D strain of wild-type reovirus (Reolysin^®^; Oncolytics Biotech, Calgary, Canada) has been tested in various clinical trials. These studies have shown the relative safety but varying response rates of reovirus-based therapy in patients with melanoma, malignant glioma, breast cancer, pancreatic adenocarcinoma, colorectal carcinoma, or other types of tumors (19–24).

It has been suggested that the varied response to reovirus-based therapy could be an indication that oncolytic virus-based treatments can reshape the tumor microenvironment (TME). It was later shown that oncolytic viral-based treatments can increase proinflammatory cytokines in the TME, enhance the cytotoxicity of innate immune cells, and augment antigen presentation and adaptive immune responses. For example, in a study by Muller et al., reovirus treatment increased the number of NK and cytotoxic T cells, which also exhibited a stronger activated phenotype. In addition, reovirus treatment enhanced the memory response of the immune system (25). Altogether, it has been shown that reovirus significantly alters the immune response towards the tumor (22, 25–28).

Natural killer (NK) cells are innate lymphoid cells which are considered to be a key factor in the defense against tumor transformation and viral infections, as they can identify a wide range of patterns associated with “danger,” and unleash a cytotoxic response towards those patterns (29). NK cell cytotoxicity is regulated by an array of activating and inhibitory receptors (30), with the NKG2D receptor being among the most prominent activating receptors of NK cells (29, 31, 32). The NKG2D receptor binds to eight stress-induced ligands present on the cell surface, which are split into two families: the MHC class I chain-related protein (MIC) family, which includes MICA and MICB, and the UL16-binding protein (ULBP) family, which includes ULBP1-6. These ligands are overexpressed in tumor cells (33), making NKG2D-mediated recognition a prominent pathway by which NK cells identify and kill transformed cells (34). Moreover, because the expression of NKG2D ligands also enhances the cytotoxic activity of NK cells towards virus-infected cells, different viruses have developed unique mechanisms to manipulate the expression of stress-induced ligands to evade NK-mediated elimination (32, 35–39).

In an attempt to shed light on the effects of reovirus infection on the cytotoxicity of NK cells towards the infected tumor cells, we have previously shown that the NK activating receptor NKp46 can specifically recognize the σ1 protein of the reovirus and that this interaction can alter the activity of NK cells towards the infected tumor cells (40). Other studies have demonstrated that reovirus-activated NK cells together with monoclonal antibody treatment synergistically enhance their anti-tumor cytotoxicity in colorectal cancer model (41), and that reovirus enhances rituximab-mediated NK cytotoxicity against chronic lymphocytic leukemia (42). Moreover, checkpoint inhibition was shown to improve the ability of NK cells to kill reovirus-infected tumor cells (43). Our discovery that NK cells possess a receptor through which they can recognize the reovirus antigen uncovered an important mechanism by which NK cells interacts with reovirus-infected cells. However, NK cell activation is dictated by and array of activating and inhibitory (44). Therefore, we focused on the influence of reovirus infection on activation via the NKG2D receptor, as little is known about the effects of reovirus infection on the expression of the NKG2D ligands on the surface of infected tumor cells, and their possible impact on NK cell cytotoxicity. These data are of crucial importance, as reovirus-based drugs are advancing in clinical phases and are soon to be employed against a wide range of tumors. Here, we demonstrate that reovirus infection can impair the expression of NKG2D ligands in melanoma and glioblastoma cells. We further demonstrated that the changes in NKG2D ligand expression on the surface of reovirus-infected melanoma cells impair the cytotoxic activity of primary human NK cells towards malignant cells.

## Materials and methods

### Cells and media

MNT-1 (ATCC, CRL-3450), a human melanoma cell line, and U87-MG (ATCC, HTB-14), a human glioblastoma cell line, were used for reovirus infection. The cells were maintained in DMEM supplemented with 10% fetal bovine serum, 1 mM sodium pyruvate, 2 mM L-glutamine, 1 mM streptomycin, and 0.1 mM NEAA. Primary human NK cells and NK-92 cell lines were used to evaluate NK activity towards reovirus-infected cells. NK-92 cells were maintained in RPMI supplemented with 10% fetal bovine serum, 1mM sodium pyruvate, 2 mM L-glutamine, 1 mM streptomycin, 0.1 mM NEAA, and 20 ng/mL recombinant IL-2, as previously described (45). To isolate primary human NK cells, peripheral blood mononuclear cells were derived from healthy donors and purified using an NK Cell Isolation Kit (Miltenyi Biotec, 130-092-657), then maintained in mixed media of F12-DMEM and DMEM at a ratio of 1:1.3, supplied with 10% human serum, 1% sodium pyruvate, 2 mM L-glutamine, 1 mM streptomycin, 0.3 mM NEAA, 100 U/mL recombinant IL-2, and 10 ng/mL recombinant IL-15. The percentage of purified NK cells was verified using anti-CD56 (BioLegend, 36503) and anti-CD3 (Thermo Fisher Scientific, 56-0038-82) antibodies, in which the CD56^+^CD3^-^ population was measured. RKO cells were used to evaluate NKG2D ligand-shedding. B16 murine melanoma cell line was used for evaluation of mouse NKG2D-Ig binding to reovirus-infected cells. All the cells were maintained at 37°C with 5% CO_2_.

### Reovirus infection and degradation inhibitors

Reovirus 3 strain Dearing (ATCC, VR824) was propagated as previously described (46), and plaque assays were performed on MNT-1 cells to determine the concentration of plaque-forming units (PFU) of the propagated reovirus stock (47). MNT1 and U87-MG cells were seeded in a six-well plate at a density of 3x10^5^ cells per well and infected with the reovirus in 0.6mL of growth media. MOI is listed in the figure legend of each figure. The cells were then incubated with the virus for 1 hour (hr) at room temperature. Following incubation, 0.9mL of growth media was added to the cells, and the cells were incubated at 37°C with 5% CO_2_. In experiments that required proteasomal or lysosomal blockers, cells were infected as described above, followed by exposure to 100 µM chloroquine (Sigma-Aldrich) or 10 µM MG132 (Sigma-Aldrich, 474790) at 24 hours (hrs) post-infection. The analyses were performed 48 hrs post-infection. In the kinetic experiments, the analyses were performed at 0, 6, 12, 24, 36, 48, and 60 hrs following infection. For UV-inactivation, viruses were irradiated with 254 nm UV light for 30 minutes, and inactivation of the viruses was confirmed by plaque assays.

### Antibodies and fusion proteins

The following primary antibodies were used for flow cytometry: anti-MICA (R&D, MAB1300), anti-MICB (R&D, MAB1599), anti-ULBP1 (R&D, MAB1380), anti-ULBP2 (R&D, MAB1298, with cross reactivity to ULBP5 and ULBP6), anti-ULBP3 (R&D, MAB1517), anti-GLUT-1 (abcam, Ab150299), anti-IFNƔ (R&D, MAB285), anti-CD56 (BioLegend, 36503), anti-CD3 (ThermoFisher Scientific, 56-0038-82), anti-σ1 (Sigma-Aldrich, MAB994-I-25UG), anti-CD4 (BioLegend, 344604), anti-CRT (Invitrogen, 902A), anti-ERp57 (BioLegend, 937301) and anti-PVR (BioLegend, 337602). Additionally, anti-KIRL2D3, murine anti-NKG2D, and anti-NKP46 antibodies were purified from hybridomas as previously described (48). These primary antibodies were stained with AF448-coupled donkey anti-mouse IgG (Jackson ImmunoResearch, 715-545-151) or APC-coupled goat anti-mouse IgG (Thermo Fisher Scientific, 31981). Murine NKG2D-Ig and human NKG2D-Ig fusion protein (R&D, 1299-NK) were followed by secondary staining with PE-coupled F(ab’)₂ fragment goat anti-human IgG (Jackson ImmunoResearch 109-116-088). Additionally, a Fixable Viability Dye eFluor™ 780 (FVD) (Thermo Fisher Scientific, 65-0865-14) was included in the flow cytometry staining to exclude the dead population.

### Flow cytometry

MNT-1, U87-MG, NK-92, or primary NK cells were harvested and washed twice in PBSx1 supplemented with 1% bovine serum albumin (FACS media) and then incubated for 40 min on ice with FACS media containing primary antibody diluted as recommended by the manufacturer. The cells were then washed with FACS media and further incubated for 40 min on ice with FACS media supplied with 0.5% secondary antibody and 0.1% FVD (ThermoFisher Scientific, 65-0865-14). Finally, the cells were washed twice and read using an LSRFortessa flow cytometer (BD Biosciences) or through an AriaIII flow cytometer (BD Biosciences). Intracellular staining of cells was performed using the Fixation/Permeabilization kit (BD Biosciences, 554714) or with the eBioscience™ Intracellular Fixation & Permeabilization Buffer Set (ThermoFisher Scientific, 88-8824-00), according to the manufacturer’s instructions. Primary antibodies were incubated with the cells for 1 hr on ice. Murine NKG2D-Ig and human NKG2D-Ig were incubated with the cells for 90 minutes. For intra-cellular FACS staining of CRT, ERp57 and GLUT-1 in MNT-1 and U87-MG cell lines, the cells were fixed using FIX & PERM Cell Fixation and Cell Permeabilization Kit (Invitrogen). Data were analyzed using the BD FACSDIVA software and further analyzed using FlowJo_10.8.1. All of our FACS analysis were done only on live cells that were stained negatively to FVD, in order to minimize the effects of eminent cell death caused by the reovirus. In all experiments, the background binding was measured through incubating unstained cells with the relevant secondary Ab.

### Generating MNT-1-CD4 and U87-CD4 cells

Lentiviruses expressing human CD4 were generated by transfecting 293T (ATCC, CRL-3216) cells with 6µg psPAX2 plasmid (Addgene, 12260), 4µg pMD2.G plasmid (Addgene,12259) and 4µg lentiMPH v2 plasmid (Addgene, 89308) containing the CD4 gene inserted at positions 7654 and 9085. Then, 0.6x10^6^ cells of MNT-1 or U87-MG cell lines were infected with CD4-lentivirus, followed by positive selection through growth media containing hygromycin B (InVivoGen, ant-hg) at a concentration of 500µg/mL. The selection media was replaced every 2 days for a period of 7 days. After selection, CD4^+^ cells were sorted using FACSAria (BD Biosciences) and stained with anti-CD4 (BioLegend, 344604) for further purification.

### Ligand shedding and ELISA

3x10^5^ MNT-1 cells were seeded in a six-well plate and infected as described above. Media from reovirus-infected and mock-infected wells were collected 48 hrs post-infection. To remove cell debris, the samples were centrifuged for 5 min at a speed of 350 rcf. The media was then concentrated to a final volume of 200 µL using Amicon Ultra centrifuge tubes (Sigma-Aldrich) and centrifuged for 30 min at 4000 rpm. For MICA and MICB testing, tubes with a molecular weight cut-off (MWCO) of 30 kDa were used (Sigma-Aldrich, UFC-9030). For ULBP2 and ULBP3 testing samples, tubes with a MWCO of 10 kDa were used (Sigma-Aldrich, UFC-8010).

As a positive control, we used RKO cells overexpressing MICA*008, which was generously donated by Prof. Ofer Mandelboim (36). 0.9x10^6^ RKO cells were treated with 2 units of phosphoinositide-specific phospholipase C (PI-PLC) (Sigma-Aldrich, P5542) for 4 h, in 600 µL of buffer prepared according to the manufacturer’s instructions. Finally, the cells were centrifuged at 350 rpm to remove cell debris, and the supernatant was collected and aliquoted into three 200 µL samples.

The concentrated media samples and the positive control were plated on an ELISA plate and left to coat overnight. The following day, the wells were washed twice with PBSx1 containing 0.05% TWEEN20 (Sigma-Aldrich, p9416; Sigma-Aldrich). They were then blocked in PBSx1 containing 2% dry milk (Sigma-Aldrich, 70166-500). Primary antibodies (R&D, MAB1300, MAB1599, MAB1298, MAB1517) were added to the wells at the concentration of 2 µg/mL and incubated for 2.5 hrs. The wells were then washed six times with PBSx1 containing 0.05% TWEEN20. The secondary HRP-conjugated anti-mouse antibody was added to the wells at a concentration of 2 µg/mL and incubated for 1.5 hrs, followed by 6 washed with PBSx1 containing 0.05% TWEEN20. Finally, 100 µL TMB Single Solution (Life Technologies, 02049211-7) was added to the wells and incubated for 12 min before being read in Epoch (BioTek) at 605 nm.

### Quantitative polymerase chain reaction analysis

MNT-1 cells, U87-MG cells, primary human NK cells and NK-92 cells were incubated with reovirus as previously described. After 48 hrs, cellular RNA was isolated using the Total RNA Mini kit (Geneaid, RBD050), followed by cDNA synthesis using an iScript cDNA synthesis kit (BioRad, 1708890). Finally, qPCR was performed using the PowerTrack SYBR Green Master Mix (Thermo Fisher Scientific, A46109). All kits were used according to the manufacturer’s instructions. The qPCR reaction was read using a CFX Connect Real-Time System (Bio-Rad). The primers used in the experiment were: GAPDH forward primer GATTCCACCCATGGCAAATTC, reverse primer GGCAGAGATGATGACCCTTT. Actin forward primer AAGACCTGTACGCCAACAC, reverse primer GTCATACTCCTGCTTGCTGAT. MICA forward primer ATCTGTGCAGTCAGGGTTTC, reverse primer GCCTTCTTTCTGGTCCTTGATA. MICB forward primer ATCTGTGCAGTCAGGGTTTC, reverse primer CCTCCTTCTGGTCCTTGATATG. ULBP2 forward primer CTGGAGCCAGAAAGATGAAAGA, reverse primer ATGATGAGGAGGCAGCAAAG. ULBP3 forward primer GGATGAAAGAGAAGTGGGAGAAG, and reverse primer GGATGAAGCAGAGGATGATGAG. CRT forward primer CCTCACCCCTGGTTCTCATC, reverse primer GAGGAAGAACGGGGCTCTTC. ERp57 forward primer GCTGCAGCTACCAGATTAAAAGG, reverse primer GACCAGCTTCAGTGCCTCTC. S4 forward primer CTGGCTTCAGGTTGACCCAA, reverse primer TGGGTTTGACGAGCATCTCC. M3 forward primer ATGATGGCAGGATGGCTGAC, reverse primer AGCGTTGTCCCGATCTTTGA. L3 forward primer GAGACTGGACTGATGGTGCC, TTTCTTCAAGCCAGGCGGAT reverse primer. All samples were tested in triplicates.

### Electroporation

The S1-4, M1-3 and L1-3 viral genes were inserted into pAAV-DYRK4-2A-GFP (Addgene, 118288) at positions 1323 and 3111. MNT-1 cells were harvested, washed twice, and resuspended at a density of 10^7^ cells/mL in Opti-MEM. A total of 10^6^ cells were mixed with 10µg of the target plasmid, followed by electroporation with NEPA21 (Almog Diagnostics) using appropriate cuvettes (Almog Diagnostics, ec-002). Poring pulse: voltage 210V, pulse length 3ms, pulse interval 50ms, D. rate 10%, positive polarity, two pulses. Transfer pulse: voltage 20V, pulse length 50ms, pulse interval 50ms, D. rate 40%, positive/negative polarity, five pulses. After electroporation (EP), the cells were cultured in growth media (1.5mL) for 72 hrs until further analysis. All the plasmids used in the EP experiments were sequenced. The prevalence and the NKG2D ligand-phenotype of GFP^+^ cells were evaluated using flow cytometry.

### NK cytotoxicity assay

3x10^5^ MNT-1 cells were infected with 3x10^7^ PFU of reovirus for 1 hr at room temperature, then incubated at 37˚C. At 48 hrs of infection, the cells were harvested and labelled with CFSE (ThermoFisher Scientific, C34570) according to the manufacturer’s instructions. They were then cocultured with purified primary NK cells for 4 hrs at effector-to-target (E:T) ratios of: 10:1, 5:1, 2.5:1, or 1:1. NK cells were adjusted to the number of target cells. To assess spontaneous lysis of cells, samples containing target cells only were included for both the infected and uninfected groups. The lysis of target cells was assessed by staining each sample with 0.01% fixable viability dye (FVD), and was measured by flow cytometer. Following doublets discrimination, the percentage of the specific lysed population was gated as %CFSE^+^FVD^+^ out of the CFSE^+^ population. NK cell specific cytotoxicity was calculated by following formula . All tested groups, including different E:T ratios, were done in triplicate. In assays with NKG2D receptor blocking, prior to coculturing with target-cells, NK cells were washed twice in PBSx1 and incubated on ice for 30 min with anti-NKG2D (R&D, MAB139) at the concentration of 10µg/mL. The cells were then washed twice in PBSx1 and cocultured as described above.

### IFNƔ quantification assay

NK-92 cells were cocultured with reovirus-infected or mock-infected target MNT-1 cells for 20 hrs at an effector-to-target (E:T) ratio of 2.5:1, followed by 4 hrs of exposure to Brefeldin A (Thermo Fisher Scientific, 00-4506-51) and monensin (ThermoFisher Scientific, 00-4505-51). The cells were stained with FVD, anti-CD3 and anti-CD56. They were then fixed and stained for IFNƔ. The results were analyzed using flow cytometry. Following doublets discrimination, the FVD^-^CD3^-^CD56^+^ population was further analyzed for the expression of IFNƔ. PMA and Ionomycin - eBioscience™ Cell Stimulation Cocktail (500X), (ThermoFisher Scientific 00-4970-93) was used to stimulate NK cells as IFNƔ^+^ control. All tests were performed in triplicate.

### CFSE proliferation assay

NK-92 cells were stained with CFSE according to the manufacturer’s instructions, followed by coculture with infected or uninfected target cells for 96 hrs at an effector-to-target (E:T) ratio of 2.5:1. NK cell counts were adjusted according to the number of target cells. The cells were stained with FVD, anti-CD56 and anti-CD3. Following doublet discrimination, the FVD^-^CD3^-^CD56^+^ population was further assessed by FlowJo proliferation analysis, using CFSE as a proliferation marker. A group of unstimulated NK-92 cells cultured alone was used as a control. All tests were performed in triplicate.

### Statistical analysis

Normal distribution was tested using the Shapiro-Wilk test. Experiments wherein normal distribution was determined, statistical significance was calculated by one-tailed unpaired Student’s t-test, two-tailed unpaired Student’s t-test, or two-way ANOVA followed by either Sidak’s or Tukey’s test. Multiple t-tests were corrected with a false discovery rate (FDR) of 0.01. Experiments wherein normal distribution was not likely, Kruskall-Wallis test followed by Mann-Whitney tests were used. The legend of each figure addresses the specified statistical test used. Statistical tests were performed using GraphPad Prism. Quantitative PCR analysis and Cq calculations were performed using the CFX Manager software 3.1 (BioRad). Data are presented as the mean ± SEM. A value of p> 0.05 was deemed not statistically significant.

## Results

### Reovirus infection reduces the expression of NKG2D ligands on the surface of tumor cells

We and others have previously demonstrated that NK cells can directly eliminate reovirus-infected tumor cells (40–42). Here, we sought to evaluate whether the reovirus has developed mechanisms to impair NK cell cytotoxicity towards infected cells. We analyzed the levels of NKG2D ligands on the surface of reovirus-infected tumor cells, as NKG2D is a dominant NK cell receptor that augments NK-cell anti-viral activity (29, 31, 32). We infected the human melanoma cell line MNT-1, and human glioblastoma cell line U87-MG with reovirus T3D. Both of these cell lines were shown to be permissive to reovirus infection and have an aberrant RAS/PI3K signaling (49–52). Infection was conducted using 3x10^7^ PFU of the reovirus to infect 3x10^5^ cells of MNT-1 melanoma and U87-MG glioblastoma cell lines (MOI=100). Infection analyses were performed only on the live population (**Figure S1A**). High infection rates were observed in the live population in both cell lines, with 93.9% of U87-MG cells (**Figure S1B**) and 82.7% of MNT-1 cells expressing the σ1 protein of the reovirus (**Figure S1C**). The same gating strategy was applied in all the experiments in this study (**Figure S1A**).

**Figure 1:**
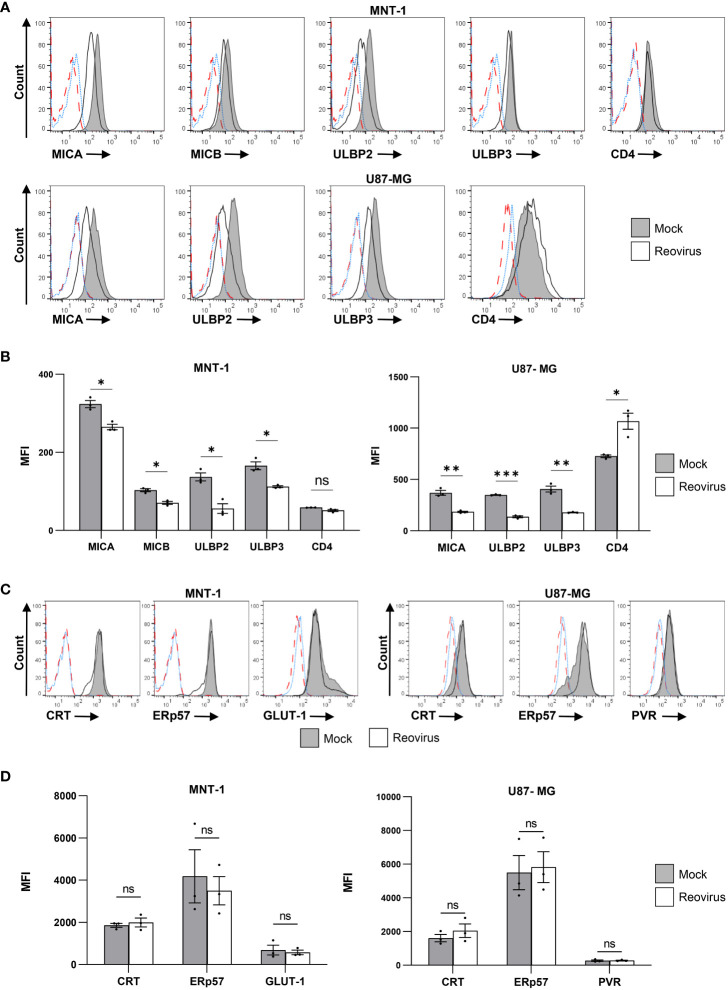
Reovirus infection reduces the expression of NKG2D ligands on the surface of infected cells. **(A)** FACS staining for NKG2D-ligands (MICA, MICB, ULBP2, ULBP3) and for human CD4 in MNT-1 cell line and in U87-MG cell line 48 hrs post infection. CD4 staining was done on transduced CD4^+^ cells, NKG2D ligands staining was done on WT cell lines. Grey histogram depicts mock-infected cells, black line depicts reovirus infected cells, red dashed-line depicts the background staining of mock-infected cells, blue dotted-line depicts the background staining of reovirus-infected cells. Shown is 1 representative staining out of 3 that were performed. MOI=100 was used in this experiment. **(B)** Summary of the geometric mean fluoresce intensity (MFI) of the staining shown in **(A)**. Grey columns depict mock-infected cells, white columns depict reovirus-infected cells. Shown is a summary of 3 experiments. Two-tailed unpaired Student’s t test was used to determine statistical significance. FDR of 0.01 was applied. *p<0.05, **p<0.01, ***p<0.005, ****p<0.0001. Each dot on the plot depicts the result of an independent experiment. Data is presented as mean ± SEM. **(C)** Intra cellular FACS staining for CRT, ERp57, PVR and GLUT-1 in the MNT-1 cell line and the U87-MG cell line. Grey histogram depicts mock-infected cells, black line depicts reovirus infected cells, red dashed-line depicts the background staining of mock-infected cells, blue dotted-line depicts the background staining of reovirus-infected cells. Shown is 1 representative staining out of 3 that were performed. MOI=100 was used in this experiment. **(D)** Summary of the MFI of the staining shown in **(C)**. Grey columns depict mock-infected cells, white columns depict reovirus-infected cells. Shown is a summary of 3 experiments. Two-tailed unpaired Student’s t test was used to determine statistical significance. FDR of 0.01 was applied. *p<0.05, **p<0.01, ***p<0.005, ****p<0.0001. Each dot on the plot depicts the result of an independent experiment. Data is presented as mean ± SEM.

Next, we evaluated the expression levels of the NKG2D ligands MICA, MICB and ULBP1-3 at 48 hrs post-infection (**Figures 1A, B**). ULBP1 was not detected on the surface of either cell line (**Figure S1D**). Downregulation of the surface expression of MICA, ULBP2, and ULBP3 was observed in both MNT-1 and U87-MG cells following reovirus infection (**Figures 1A, B**). MICB was also downregulated in reovirus-infected MNT-1 (**Figure 1A**) cells compared to mock-infected cells, but was not expressed on the surface of U87-MG cells (**Figure S1E**). As control, U87-MG and MNT-1 cells were engineered to stably express CD4 on their surface, and no significant changes were seen in CD4 surface expression upon reovirus infection (**Figures 1A, B**).

To validate that the observed downregulation was not due to general interference with host protein expression, we tested the surface levels of four endogenously expressed proteins: calreticulin (CRT), ER protein 57 (ERp57), the poliovirus receptor (PVR) and the glucose transporter 1 (GLUT-1). The surface levels of CRT were of particular interest, as CRT, was recently identified as a novel ligand for the NK activating receptor NKp46 (53). Thus, CRT provided a control with comparable biology in terms of function, target and turnover time (53, 54). We observed no significant differences in the expression of CRT, ERp57, PVR or GLUT-1 in mock-infected cells compared to reovirus-infected cells (**Figures 1C, D**). Furthermore, a previous study by S. Schmechel et al. has demonstrated that the reovirus T3D does not impair cellular protein translation in infected cells (55).

### Reovirus infection impairs the binding of NKG2D-Ig to infected-tumor cells

Changes in the surface expression of NKG2D ligands have been shown to significantly alter NK cell cytotoxicity to tumor cells and virus-infected cells (32). Thus, we wanted to further analyze the kinetics of NKG2D ligand downregulation following reovirus infection, in order to gain a thorough understanding of how reovirus infection of tumor cells might affect NKG2D-binding in the tumor microenvironment. Moreover, we have increased the reovirus MOI (MOI=200) to evaluate if the level of the NKG2D ligand downregulation is dependent on the amount of virus that is used for infection. Since MICB was expressed only in MNT-1 cells (**Figure S1E**), we focused our analysis on this cell line. To study the downregulation kinetics, we selected seven representative time points: 0, 6, 12, 24, 36, 48, and 60 hrs post-infection. Later time points were limited by extensive cell death and by a small live population in the reovirus-infected group, which prevented an accurate analysis (**Figure S2A**).

**Figure 2:**
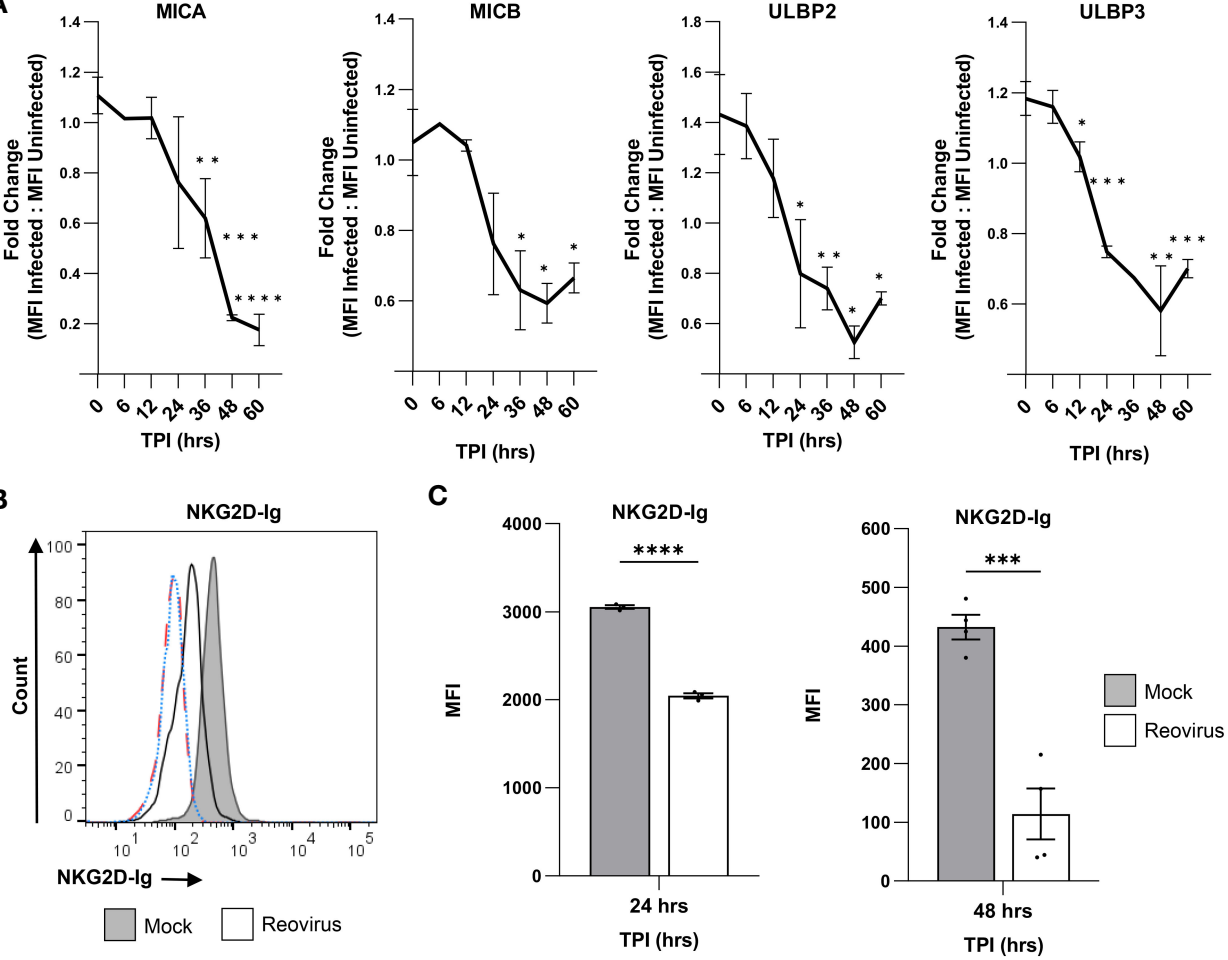
Reovirus infection impairs the binding of NKG2D-Ig to infected cells. **(A)** The kinetics of down-regulation of NKG2D ligands (MICA, MICB, ULBP2 and ULBP3) in reovirus-infected MNT-1 cells compared to mock-infected MNT-1 cells. The y axis shows fold change of MFI of reovirus-infected cells divided by MFI of mock-infected cells. The x axis shows time post infection (TPI) in hrs. Shown are 3 pooled experiments in each time point. Statistical significance was tested in comparison to time point 0 hrs post infection. MOI=200 was used in these experiments. Two-tailed unpaired Student’s t test was used to determine statistical significance. *p<0.05, **p<0.01, ***p<0.005, ****p<0.0001. Data is presented as mean ± SEM. **(B)** FACS staining with NKG2D-Ig fusion protein after incubation with mock-infected and reovirus-infected MNT-1 cells, 48 hrs post infection. Grey histogram depicts mock-infected cells, black line depicts reovirus infected cells, red dashed-line depicts the background staining of mock-infected cells, blue dotted-line depicts the background staining of reovirus-infected cells. Shown is 1 representative staining out of 3 that were performed. MOI=100 was used in these experiments. **(C)** Summary of the MFI of the staining with human NKG2D-Ig at 24 hrs post infection and at 48 hrs post infection. The grey columns depict mock-infected cells and the white columns depicts reovirus-infected cells. Shown is a summary of 3 experiments in each time point. MOI=100 was used in these experiments. Two-tailed unpaired Student’s t test was used to determine statistical significance. *p<0.05, **p<0.01, ***p<0.005 ****p<0.0001. Each dot on the plot depicts the result of an independent experiment. Data is presented as mean ± SEM.

We observed a downregulation in MICA at 36 hrs post-infection, with a decreasing trend at the following time points (**Figure 2A**). Similarly, MICB was first downregulated at 36 hrs post-infection; however, we did not observe a further decrease in expression at later time points (**Figure 2A**). ULBP2 and ULBP3 were downregulated at earlier stages of infection. ULBP2 expression was reduced at 24 hrs post-infection (**Figure 2A**), and ULBP3 expression was reduced as early as 12 hrs post-infection (**Figure 2A**). In all tested ligands, we observed no time point at which the expression of the ligands was elevated in comparison to the time point 0 hrs of infection (**Figure 2A**). Finally, the increased amount of reovirus (MOI=200) that was used in these experiments augmented the downregulation of all NKG2D ligands compared to infection with reovirus at MOI=100 (**Figures 1**, **2**).

Next, we tested whether the reovirus-mediated downregulation of NKG2D ligands would impair the recognition of infected tumor cells by the NKG2D receptor. To this end, we stained mock-infected and reovirus-infected MNT-1 cells with a fusion protein composed of the extracellular portion of the human NKG2D receptor, fused to the Fc portion of human IgG1 (NKG2D-Ig). Notably, a significant reduction in the binding of NKG2D-Ig to reovirus-infected cells was observed in comparison with mock-infected cells 24 hrs and 48 hrs post-infection (**Figures 2C**, **C**, **S2B**, **C**). To validate this result, we repeated the experiment using the U87-MG glioblastoma cell line and obtained concurring results (**Figure S2D**). Overall, this data together with the kinetics of the NKG2D-ligands downregulation (**Figure 2**) indicated that 24 hrs and 48 hrs after tumor cell infection, the NKG2D recognition of tumor cells is diminished.

### The translation of MICA, MICB and ULBP3 in tumor cells is impaired following reovirus infection

Viruses have developed diverse mechanisms that hinder the expression of NKG2D ligands in infected cells to evade NK cell-mediated elimination. This includes interference with NKG2D ligand transcription, rapid degradation of mRNA, decreased protein synthesis, increased protein degradation, and shedding of ligands from the cell surface (56).

**Figure 3:**
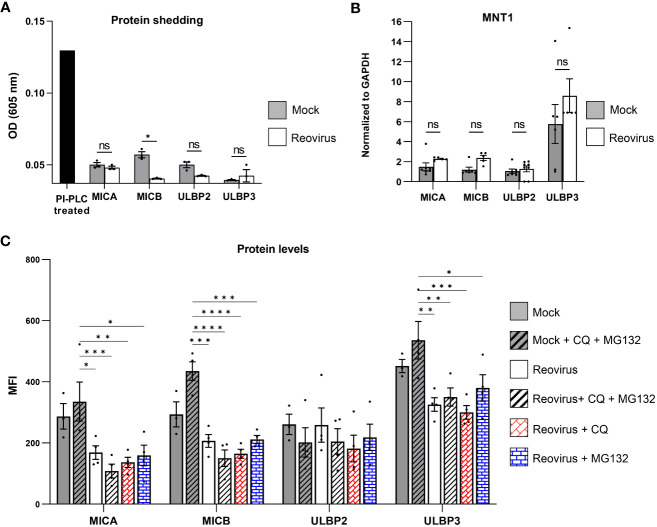
The Mechanism of NKG2D ligands downregulation in reovirus-infected cells. **(A)** ELISA analysis of growth media of mock-infected or reovirus-infected cells at 48 hrs post infection. A sample of RKO-MICA*008 cells treated with PI-PLC enzyme was included as a positive control. Shown 1 out of 3 independent experiments. The black column depicts the positive control of PI-PLC treatment, grey columns depict the mock-infected group, the white columns depict reovirus-infected group. MOI=100 was used in these experiments. Two-tailed unpaired Student’s t test was used to determine statistical significance. FDR of 0.01 was applied. *p<0.05, **p<0.01, ***p<0.005, ****p<0.0001. *p<0.05, **p<0.01, ***p<0.005, ****p<0.0001. Each dot on the plot depicts the result of an independent experiment. Data is presented as mean ± SEM. **(B)** Analysis of quantitative polymerase chain reaction (qPCR) of the NKG2D ligands MICA, MICB, ULBP2 and ULBP3 normalized to glyceraldehyde-3-phosphate dehydrogenase (GAPDH) at 48 hrs post infection. The grey columns depict mock-infected cells, the white columns depict reovirus-infected cells. Shown is a summary of 3 independent experiments. MOI=100 was used in these experiments. Mann-Whitney tests were used to determine statistical significance. *p<0.05, **p<0.01, ***p<0.005, ****p<0.0001. Each dot on the plot depicts the result of an independent experiment. Data is presented as mean ± SEM. **(C)** Expression of NKG2D-ligands (MICA, MICB, ULBP2, ULBP3) in mock-infected or reovirus-infected MNT-1 cells at 48 hrs post infection. Grey columns depict mock-infected cells, white columns depict reovirus-infected cells. The y axis depicts the MFI. The x axis depicts NKG2D-ligands, grouped by treatment type. Mock-infected and reovirus-infected cells, were treated with mock, 100µM of chloroquine (CQ), 10µM of MG132, or a combination of both. Shown is pooled data from 4 independent experiments. MOI=100 was used in these experiments. Two-way ANOVA followed by Tukey’s test were used to determine statistical significance. *p<0.05, **p<0.01, ***p<0.005, ****p<0.0001. Each dot on the plot depicts the result of an independent experiment. Data is presented as mean ± SEM.

To uncover the mechanism by which reovirus infection leads to the reduced expression of MICA, MICB, ULBP2, and ULBP3 in infected cells, we first evaluated their shedding from the cell surface. ELISA was performed on growth media collected from reovirus-infected and mock-infected MNT-1 cells 48 hrs post-infection. As a positive control, we used RKO cells expressing the glycosylphosphatidylinositol (GPI)-anchored allele of MICA, MICA*008 (36). Upon incubation with PI-PLC, the PI link is cleaved, leading to the release of MICA*008 into the media. The shedding of MICA*008 was validated using FACS staining (**Figure S2E**). In contrast to the positive control, in which high optical density (OD) was observed, low amounts of NKG2D ligands were detected in the media samples of reovirus-infected and mock-infected MNT-1 cells. Shedding of MICA, ULBP2, and ULBP3 was not enhanced post-infection, whereas shedding of MICB was reduced (**Figure 3A**). Altogether, we concluded that the downregulation of NKG2D ligands in infected tumor cells is not due to shedding from the cell membrane.

Next, we tested other mechanisms by which the reovirus could manipulate the NKG2D ligand-expression. We performed quantitative polymerase chain reaction (qPCR) on mRNA derived from mock-infected or reovirus-infected MNT-1 cells 48 hrs post-infection. The results were normalized to GAPDH levels, as it had stable expression-levels following reovirus infection (**Figure S2F**). In contrast to the reduced levels of these proteins on the cell surface of the infected cells, no effect on the mRNA levels of MICA, MICB, ULBP2, and ULBP3 was observed following infection (**Figure 3B**), or on the mRNA levels of CRT and ERp57 (**Figure S2G**), indicating that the impaired expression of MICA, MICB, ULBP2, and ULBP3 in reovirus-infected cells was not due to reduced transcription levels of these NKG2D ligands.

Thus, we further tested whether the intracellular protein levels of MICA, MICB, ULBP2, and ULBP3 were altered upon reovirus infection. We analyzed the intracellular levels of NKG2D ligands in mock-infected and reovirus-infected cells at 48 hrs post-infection. We observed reduced levels of MICA, MICB, and ULBP3 in infected cells, but no significant changes in ULBP2 levels (**Figure S2H**).

Furthermore, to determine whether the reduced protein levels of MICA, MICB, and ULBP3 were due to impaired translation or increased protein degradation, we treated mock-infected and reovirus-infected cells with protein degradation inhibitors. Reovirus-infected cells were treated with either the lysosomal blocker chloroquine (CQ), proteasome blocker MG132, or a combination of both blockers. Applying those treatments 24 hrs post infection did not alter the viability of cells, suggesting that, at this time point, CQ and MG132 do not inhibit the release of the viral core to the cell (**Figure S3B**). Intracellular protein levels of MICA, MICB, ULBP2, and ULBP3 were tested 48 hrs post-infection. In accordance with our previous results, which demonstrated that ULBP2 protein levels were not affected by reovirus infection (**Figure S2H**), protein degradation inhibitors did not lead to significant changes in ULBP2 expression, indicating that the downregulation of ULBP2 is not dependent on induced degradation or impaired translation (**Figure 3C**). Notably, protein degradation inhibitors failed to rescue the expression of MICA, MICB, and ULBP3 (**Figure 3C**), indicating that the reduced protein levels of MICA, MICB, and ULBP3 in the infected tumor cells were due to the impaired translation of these ligands, and not due to increased protein degradation.

Moreover, to test the prospect that the downregulation in MICA, MICB, and ULBP3 is due to a cellular response triggered by innate immune sensing of RNA viruses, we have incubated MNT-1 cells and U87-MG cells with a UV-inactivated reovirus and followed the surface expression of the NKG2D-ligands 48 hrs after the initial incubation. No consistent reduction in the surface expression of MICA, MICB, and ULBP3 was observed following exposure of the cells to the UV-inactivated reovirus (**Figure S3A**), as we have previously shown (40).

To identify the viral gene responsible for the diminished translation of MICA, MICB and ULBP3, and the gene responsible for the reduced surface levels of ULBP2, we transferred viral genes into cells using electroporation (EP). To ensure the accuracy of results, we applied the T2A-GFP system, which allows reliable analysis of cells positive for the viral gene in prospect, as its expression correlates with the GFP^+^ signal (**Figures S3C, D**). Of note, we tested whether EP alters the expression of NKG2D ligands and concluded that the expression remains stable, as levels of NKG2D ligands on the surface of MNT-1 cells were not affected after EP (**Figure S3E**). Additionally, we infected MNT-1 cells that were previously subjected to EP with reovirus and found that the EP did not interfere with the downregulation of NKG2D ligands post reovirus infection (**Figure S3F**).

Initially, we focused on the viral proteins that were previously described to have RNA-binding properties, proteins that facilitate the formation of viral factories, or proteins that have been reported to alter protein translation. Of the 10 viral genes that form the reovirus genome, six genes have been described to harbor at least one of these properties: S2, S3, S4, M1, M3, and L3 (57–61). After cloning these viral genes into the T2A-GFP plasmid, the plasmid was electroporated into MNT-1 cells and the GFP^+^ population was evaluated. The transfection efficacy of all plasmids was sufficient, reaching 75.3% (**Figure S3D**). The surface levels of NKG2D ligands were analyzed in GFP^+^ cells compared to the GFP^-^ population in the same sample, as well as in a sample electroporated with a mock GFP plasmid without an inserted viral gene. However, none of these genes induced downregulation of NKG2D ligands 72 hrs after EP (**Figure S4**).

Next, we investigated the possibility that genes that do not typically interfere with protein expression have evolved to specifically inhibit the translation of NKG2D ligands. S1 was further investigated, as it has been suggested to act as a cofactor in the process of viral protein translation in the reovirus strain type 1 and could have a similar activity in the reovirus T3D used in this study (62). Additionally, L2 was analyzed because of its mRNA-capping properties (63). The M2 gene, which produces the outer capsid protein, and the L1 gene, which produces RNA-dependent RNA polymerase, were also included in our analysis. Neither of these two proteins was described to affect translation in infected cells, yet they were included to provide a complete evaluation of all reovirus proteins. Using the same method described above, none of these genes could induce the downregulation of NKG2D ligands 72 hrs post-EP (**Figure S4**). Overall, our single-gene analysis could not identify a viral gene that leads to the same effect as the complete genome of the reovirus, indicating that it may be an effect dependent on several viral genes.

### Reovirus-mediated downregulation of NKG2D ligands diminishes NK cells activity towards infected-tumor cells

**Figure 4:**
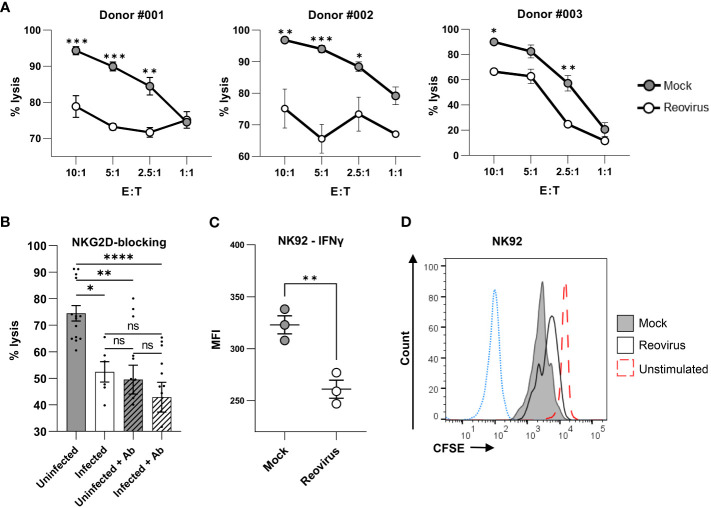
Reovirus infection impairs NK cell cytotoxicity. **(A)** NK cytotoxicity assay for mock-infected or reovirus-infected MNT-1 cells 4 hrs after coculturing with NK cells derived from 3 different donors (#001, #002, #003), at different effector to target (E:T) ratios that are shown in the x-axis. The y axis depicts cell lysis attributed to NK-cytotoxicity. Grey dots depict mock-infected cells, white dots depict reovirus-infected cells. Shown is 1 out of 3 experiments that were performed, each consisting of 3 biological triplicates, with cells derived from the 3 different donors. MOI=100 was used in these experiments. Two-way ANOVA followed by Sidak’s test were used to determine statistical significance. *p<0.05, **p<0.01, ***p<0.005, ****p<0.0001. Each dot on the plot depicts the mean. Data is presented as mean ± SEM. **(B)** NK cytotoxicity assay for mock-infected or reovirus-infected MNT-1 cells, 4 hrs post coculturing with NK cells which were incubated without antibody or with NKG2D-blocking-antibody (Ab) prior to coculturing. Coculturing was done at the E:T ratio of 5:1. Shown is summary of experiments performed with NK cells derived from 3 independent donors, each experiment consisting of biological triplicates. MOI=100 was used in these experiments. Two-way ANOVA followed by Sidak’s test were used to determine statistical significance. *p<0.05, **p<0.01, ***p<0.005, ****p<0.0001. Each dot on the plot depicts the result of an independent experiment. Data is presented as mean ± SEM. **(C)** IFNƔ production in NK-92 cells 24 hrs after coculturing with mock-infected or reovirus-infected MNT-1 cells at the E:T ratio of 2.5:1. MOI=100 was used in these experiments. Two-tailed unpaired Student’s t test was used to determine statistical significance. *p<0.05, **p<0.01, ***p<0.005, ****p<0.0001. Each dot on the plot depicts the result of an independent experiment. Data is presented as mean ± SEM. **(D)** FACS staining for CFSE in NK-92 cells 96 hrs after coculturing with mock-infected or reovirus-infected MNT-1 cells. Grey histogram depicts NK-92 cells which were incubated with mock-infected MNT-1 cells, black line depicts NK-92 cells which were incubated with reovirus-infected MNT-1 cells, red dashed-line depicts NK-92 cells which were incubated alone without stimulation, blue dotted-line depicts the background staining of NK-92. Shown is 1 representative staining out of 5. MOI=100 was used in these experiments.

NK cell cytotoxicity is controlled and modulated by an array of activating and inhibitory receptors (30). As reovirus is currently being tested in numerous clinical trials for the treatment of melanoma and other cancers, it is of special interest to evaluate whether changes in NKG2D receptor binding to reovirus-infected tumor cells can alter the function of NK cells in the tumor microenvironment. To test whether reovirus infection affects the cytotoxic activity of NK cells towards tumor cells, we purified primary human NK cells from three independent donors with variable NK cell surface receptor repertoires (**Figure S5A**). We then performed an NK cytotoxicity assay with purified primary human NK cells against mock- infected and reovirus-infected MNT-1 cells. Importantly, the levels of NK cytotoxicity towards tumor cells were significantly reduced following reovirus infection (**Figure 4A**). This significant reduction in cytotoxicity was observed in NK cells derived from all three donors (**Figure 4A**) despite the variability in the expression of NK receptors on the cell surface (**Figure S5A**). Pooling of data acquired from the three donors, also yielded a significant reduction in the NK-mediated cytotoxicity against reovirus-infected tumor cells (**Figure S5C**). To evaluate whether the impaired NK cell cytotoxicity towards the reovirus-infected tumor cells, is mediated by the reduced expression of NKG2D ligands, we repeated the cytotoxicity assay with primary NK cells derived from the 3 donors, while blocking the NKG2D receptor with NKG2D-blocking antibody (38). Similar to the results shown in **Figure 4A**, the cytotoxic activity of NK cells against reovirus-infected cells was diminished compared to that in mock-infected cells. However, following NKG2D receptor blockade, the killing of reovirus-infected cells was similar to that of mock-infected cells (**Figure 4B**). Taken together, these results indicate that altered expression of NKG2D ligands impairs NK cell cytotoxicity against reovirus-infected tumor cells through reduced NKG2D receptor recognition. Of note, we also tested if NK cells can be infected with reovirus in our *in vitro* experimental settings, which in turn could account for their altered cytotoxic activity. However, no infection of NK cells with reovirus was observed as tested by qPCR analysis of three different reovirus genes of RNA isolated from NK cells (**Figure S5B**).

Finally, we aimed to determine whether, in addition to the direct elimination of tumor cells, other NK cell functions are altered due to reovirus infection of target cells. These experiments were performed using the prominent human NK cell line NK-92, which is currently being tested in advanced clinical trials as a possible cellular immunotherapeutic against tumors (45, 64–66). The cytotoxicity of NK-92 against MNT-1 was validated in a cytotoxicity assay, in which cytotoxicity towards reovirus-infected cells was reduced (**Figure S5D**), consistent with the results observed in primary NK cells (**Figure 4A**). Cell proliferation and IFNγ production by NK-92 cells were analyzed following incubation with mock-infected or reovirus-infected MNT-1 cells. IFNγ production was tested 24 hrs post-coculturing, whereas proliferation was tested 96 hrs post-coculturing. We found that NK-92 cells cocultured with reovirus-infected MNT-1 cells produced less IFNγ compared to NK-92 cells cocultured with mock-infected MNT-1 cells (**Figure 4C**). Additionally, NK-92 cells which were exposed to reovirus-infected cells proliferated at a lower rate than NK-92 cells incubated with mock-infected MNT-1 cells (**Figure 4D**). Altogether, this indicates that reovirus may hinder NK-92 cell activity, which may lead to poor functionality of this cell line when used in parallel with reovirus-based treatments.

To gain further insights on whether the impaired elimination of reovirus-infected tumor cells is limiting or enhancing the tumor growth, we followed the MNT-1 cells elimination during the course of four days in the presence of either reovirus at low MOI (MOI=0.5), NK-92 cells or a combination of NK-92 and reovirus. Under these settings, the MNT-1 elimination is dominated by reovirus-mediated lysis of the tumor cells, as only 11.65% of the MNT-1 cells survived following incubation with reovirus in comparison to 64.5% survival in MNT-1 cells that were incubated with NK-92 cells (**Figure S5E**). Moreover, no significant changes were seen in the elimination of the MNT-1 cells following incubation with reovirus or a combination of reovirus and NK-92 (**Figure S5E**). Our findings indicate that the impaired elimination of reovirus-infected tumor cells by NK cells, may result in an enhanced replication of the reovirus within the tumor. Consequently, this could lead to improved tumor elimination during reovirus therapy.

The dominant effect of reovirus-mediated tumor elimination could be specific to the MOI and the experimental setting that were used in our assay, which does not reflect the tumor microenvironment in the host. Thus, we attempted to design a mouse model that will make it possible to better assess the outcome of the NKG2D ligands downregulation on reovirus-based therapy. We infected the mouse melanoma cell line B16 with reovirus and tested the binding of mouse NKG2D-Ig to the mock-infected B16 cells and the reovirus-infected B16 cells. No significant differences were observed in mouse NKG2D-Ig binding between mock-infected and reovirus-infected B16 cells (**Figure S5F**). These results reflect the poor homology of human NKG2D ligands and murine NKG2D ligands (67–70).

## Discussion

Talimogene Laherparepvec (T-VEC), which is based on herpes simplex virus (HSV-1), was the first oncolytic drug approved by the Food and Drug Administration (FDA) in 2015. Since then, oncolytic viruses have attracted much interest in oncology-related research (25). Pelareorep, a proprietary isolate of the reovirus Type 3 Dearing strain, has shown varied efficacy in treating melanoma, glioblastoma, colorectal, lung, and ovarian cancers (71). Although much of the therapeutic capacity of reoviruses is attributed to their ability to selectively replicate in and lyse RAS-transformed cells, several studies have demonstrated the ability of oncolytic viruses to trigger the host immune system against infected tumor cells, which could augment tumor elimination (22, 26–28). It was further shown that reovirus anti-tumor activity is mediated through the activation of innate and adaptive host immune systems through dendritic cells, NK cells, and effector T-cells (72, 73). NK cells are directly activated by reovirus-infected cells (40). Dendritic cells, which are activated by reovirus, can stimulate NK cell cytotoxicity (73). Moreover, reovirus infection increases the number of activated NK cells during the treatment of multiple myeloma (25). Finally, the interaction of NK cells with reovirus was also found to be important in other immune-mediated disorders, as NK cells have been shown to promote reovirus-mediated loss of tolerance to dietary antigens (74).

Our current work sheds new light on the complex nature of reovirus-NK cell interactions, as we uncovered that reovirus downregulates NKG2D ligands from infected tumor cells to impair NK cell cytotoxicity. NK cells are very heterogenic in the receptor repertoire, as receptor expression is determined in the germline, which results in differential NK cell populations even in the same individual depending on the microenvironment (75–77). Despite this heterogeneity, we demonstrated a consistent inhibition of cytotoxicity against reovirus-infected tumor cells in NK cells derived from three independent donors with different cell-surface receptor repertoires. This suggests that the immune evasion mechanism that we have uncovered has a dominant role in NK cell anti-tumor cytotoxicity. These results may partially explain the varying response rates of reovirus-based therapies observed in different clinical trials. Our findings highlight the possible therapeutic potential of combination therapy with oncolytic viruses and immune checkpoint inhibitors against various types of tumors, in which oncolytic virus treatments are used together with NK-activating antibodies.

The poor homology between human NKG2D ligands and murine NKG2D ligands (67–70) hindered us from determining whether reovirus-mediated downregulation of NKG2D ligands impairs or promotes reovirus-based cancer therapy. Moreover, our work highlights that the NK-response to reovirus infection is fundamentally different towards human or murine cell lines, as the cytotoxic activity is also dependent on the presence or absence of NKG2D ligands compared to other ligands that activates NKp46 (40). We postulate that the ability of the reovirus to restrain NK cell cytotoxicity will limit the elimination of tumor cells but will enable better spread of the reovirus in the tumor microenvironment, which could improve the oncolytic activity of virus-based treatments. Determining the accuracy of these two possibilities is critical, as it could have implications for the future usage of reovirus in the clinic and will enable better assessment of the relevance of this study for developing novel reovirus-based therapeutics. This could be addressed in future studies that will follow NK cell functionality and tumor growth in reovirus-treated cancer patients, in addition to testing the expression of NKG2D ligands in tumor biopsies prior to and following reovirus treatment. Alternatively, designing a recombinant reovirus which fails to reduce the human NKG2D ligands will enable to better evaluate the impact of the NKG2D ligands downregulation during reovirus-treatment in preclinical studies. This could be further tested and evaluated using ex-vivo tumor models that better resemble the tumor microenvironment and the anti-tumor immune responses (78). Moreover, although the antibody used in this study to stain for ULBP2 (R&D, MAB1298) was used in numerous studies to follow the expression of ULBP2 (36, 79, 80), the possible altered expression of ULBP5 and ULBP6 should be further analyzed as this antibody was reported by the vendor to also bind ULBP5 and ULBP6.

Additionally, we show reduced cytotoxicity, proliferation and IFNƔ production of NK-92 cell line after exposure to infected tumor cells. This indicates that reovirus-based therapy may interfere with the communication between NK cells and neighboring immune cells in the tumor microenvironment. Similarly, the NK-92 cell line is used to generate NK-based cellular immunotherapies, which are currently being tested in clinical trials (64–66). Our results suggest that NK-92-based interventions may be affected by reovirus-based treatment.

Importantly, our single gene analysis could not detect a single viral gene that induces the downregulation of the NKG2D ligand in transduced tumor cells. This may indicate that the downregulation is mediated through the cooperative function of several viral genes, as was previously shown for human cytomegalovirus (HCMV) (81). Studying the mechanism of NKG2D ligand downregulation and identifying the viral and cellular proteins that mediate this effect could be further studied using reverse genetics systems that have been developed for engineering recombinant reoviruses (82). Moreover, we showed that the downregulation mechanism of NKG2D ligands occurs through interference with translation. Finally, as shedding of NKG2D ligands is a well-described immune evasion mechanism of tumor cells (56) we also tested MICB shedding, which was reduced after reovirus infection. Our results suggest that reovirus-based therapies may be of special interest in tumors with high shedding of NKG2D ligands.

In summary, we report a novel NK-evasion mechanism of reovirus, which could have implications for future reovirus-based therapeutics.

## Data availability statement

The original contributions presented in the study are included in the article/**Supplementary Material**. Further inquiries can be directed to the corresponding author.

## Ethics statement

Ethical approval was not required for the studies on humans in accordance with the local legislation and institutional requirements because only commercially available established cell lines were used. Ethical approval was not required for the studies on animals in accordance with the local legislation and institutional requirements because only commercially available established cell lines were used.

## Author contributions

We, the authors of this research, state that the collection, processing, and presentation of data, were conducted according to the ethical standards of the academic world. YB-O is the corresponding author. RK conducted the experiments in this work. JZ, TL and SJ helped with the NK cytotoxicity assay, proliferation assay and IFNgamma assay. All work performed on NK cells, was done with their generous aid and guidance. SC and SD helped with cloning of plasmids used in electroporation experiments. All authors contributed to the article and approved the submitted version. 

## References

[1] Abad AT Danthi P. Recognition of reovirus RNAs by the innate immune system. Viruses (2020) 12(6):1–10. doi: 10.3390/v12060667 PMC7354570 32575691

[2] Danthi P Holm GH Stehle T Dermody TS. Reovirus receptors, cell entry, and proapoptotic signaling. Adv Exp Med Biol (2013) 790:42–71. doi: 10.1007/978-1-4614-7651-1_3 23884585 PMC4724410

[3] Chung. Reovirus infection induces apoptosis of TRAIL-resistant gastric cancer cells by down-regulation of Akt activation. Int J Oncol (2010) 36(4):1023–30. doi: 10.3892/ijo_00000583 20198349

[4] Dalet A Gatti E Pierre P. Integration of PKR-dependent translation inhibition with innate immunity is required for a coordinated anti-viral response. FEBS Lett (2015) 589(14):1539–45. doi: 10.1016/j.febslet.2015.05.006 25979169

[5] Lemay G. Synthesis and translation of viral mrna in reovirus-infected cells: Progress and remaining questions. Viruses (2018) 10(12):671. doi: 10.3390/v10120671 30486370 PMC6315682

[6] Clarke P Debiasi RL Goody R Hoyt CC Richardson-Burns S Tyler KL. Mechanisms of reovirus-induced cell death and tissue injury: role of apoptosis and virus-induced perturbation of host-cell signaling and transcription factor activation. Viral Immunol (2005) 18(1):89–115. doi: 10.1089/vim.2005.18.89 15802955 PMC2366905

[7] Wang J Yao N Hu Y Lei M Wang M Yang L. PHLDA1 promotes glioblastoma cell growth via sustaining the activation state of Ras. Cell Mol Life Sci (2022) 79(10):520. doi: 10.1007/s00018-022-04538-1 36107262 PMC11803017

[8] Steffen CL Kaya P Schaffner-Reckinger E Abankwa D. Eliminating oncogenic RAS: back to the future at the drawing board. Biochem Soc Trans (2023) 51(1):447–56. doi: 10.1042/BST20221343 PMC9987992 36688434

[9] Thumar J Shahbazian D Aziz SA Jilaveanu LB Kluger HM. MEK targeting in N-RAS mutated metastatic melanoma. Mol Cancer (2014) 13(1):45. doi: 10.1186/1476-4598-13-45 24588908 PMC3945937

[10] Fogel EJ Samouha A Goel S Maitra R. Transcriptome signature of immune cells post reovirus treatment in kras mutated colorectal cancer. Cancer Manag Res (2021) 13:6743–54. doi: 10.2147/CMAR.S324203 PMC8407676 34475783

[11] Van de Merbel AF van der Horst G van der Mark MH Bots STF van den Wollenberg DJM de Ridder CMA. Reovirus mutant jin-3 exhibits lytic and immune-stimulatory effects in preclinical human prostate cancer models. Cancer Gene Ther (2022) 29(6):793–802. doi: 10.1038/s41417-021-00360-2 34135475 PMC9209329

[12] Coffey MC Strong JE Forsyth PA K Lee PW. Reovirus therapy of tumors with activated ras pathway. Science (1998) 282:1132–334. doi: 10.1126/science.282.5392.1332 9812900

[13] Yang WQ Lun X Palmer CA Wilcox ME Muzik H Shi ZQ. Efficacy and safety evaluation of human reovirus type 3 in immunocompetent animals: racine and nonhuman primates. Clin Cancer Res (2004) 10(24):8561–76. doi: 10.1158/1078-0432.CCR-04-0940 15623640

[14] Alain T Hirasawa K Pon KJ Nishikawa SG Urbanski SJ Auer Y. Reovirus therapy of lymphoid Malignancies. Blood (2002) 100(12):4146–53. doi: 10.1182/blood-2002-02-0503 12393565

[15] Mohamed A Clements DR Gujar SA Lee PW Smiley JR Shmulevitz M. Single amino acid differences between closely related reovirus T3D lab strains alter oncolytic potency in vitro and *in vivo* . J Virol (2020) 94(4):e01688–19. doi: 10.1128/JVI.01688-19 PMC6997766 31748391

[16] Twigger K Vidal L White CL De Bono JS Bhide S Coffey M. Enhanced in *vitro* and in *vivo* cytotoxicity of combined reovirus and radiotherapy. Clin Cancer Res (2008) 14(3):912–23. doi: 10.1158/1078-0432.CCR-07-1400 18245555

[17] Heinemann L Simpson GR Boxall A Kottke T Relph KL Vile R. Synergistic effects of oncolytic reovirus and docetaxel chemotherapy in prostate cancer. BMC Cancer (2011) 11:221. doi: 10.1186/1471-2407-11-221 21645351 PMC3129324

[18] Sei S Mussio JK Yang QE Nagashima K Parchment RE Coffey MC. Synergistic antitumor activity of oncolytic reovirus and chemotherapeutic agents in non-small cell lung cancer cells. Mol Cancer (2009) 8:47. doi: 10.1186/1476-4598-8-47 19594950 PMC2723073

[19] Bernstein V Ellard SL Dent SF Tu D Mates M Dhesy-Thind SK. A randomized phase II study of weekly paclitaxel with or without pelareorep in patients with metastatic breast cancer: final analysis of Canadian Cancer Trials Group IND.213. Breast Cancer Res Treat (2018) 167(2):485–93. doi: 10.1007/s10549-017-4538-4 29027598

[20] Eissa IR Bustos-Villalobos I Ichinose T Matsumura S Naoe Y Miyajima N. The current status and future prospects of oncolytic viruses in clinical trials against melanoma, glioma, pancreatic, and breast cancers. Cancers (2018) 10(10):356. doi: 10.3390/cancers10100356 30261620 PMC6210336

[21] Gollamudi R Ghalib MH Desai KK Chaudhary I Wong B Einstein M. Intravenous administration of Reolysin^®^, a live replication competent RNA virus is safe in patients with advanced solid tumors. Invest New Drugs (2010) 28(5):641–9. doi: 10.1007/s10637-009-9279-8 PMC3851036 19572105

[22] Mahalingam D Goel S Aparo S Arora SP Noronha N Tran H. A phase ii study of pelareorep (REOLYSIN^®^) in combination with gemcitabine for patients with advanced pancreatic adenocarcinoma. Cancers (Basel) (2018) 10(6):160. doi: 10.3390/cancers10060160 29799479 PMC6025223

[23] Mahalingam D Fountzilas C Moseley J Noronha N Tran H Chakrabarty R. A phase II study of REOLYSIN^®^ (pelareorep) in combination with carboplatin and paclitaxel for patients with advanced Malignant melanoma. Cancer Chemother Pharmacol (2017) 79(4):697–703. doi: 10.1007/s00280-017-3260-6 28289863

[24] Bussiere LD Miller CL. Reovirus and the host integrated stress response: On the frontlines of the battle to survive. Viruses (2021) 13(2):200. doi: 10.3390/v13020200 33525628 PMC7910986

[25] Müller LME Migneco G Scott GB Down J King S Askar B. Reovirus-induced cell-mediated immunity for the treatment of multiple myeloma within the resistant bone marrow niche. J Immunother Cancer (2021) 9(3):e001803. doi: 10.1136/jitc-2020-001803 33741729 PMC7986878

[26] Augustine T John P Friedman T Jiffry J Guzik H Mannan R. Potentiating effect of reovirus on immune checkpoint inhibition in microsatellite stable colorectal cancer. Front Oncol (2022) 12. doi: 10.3389/fonc.2022.1018767 PMC9642964 36387154

[27] Ribas A Dummer R Puzanov I VanderWalde A Andtbacka RHI Michielin O. Oncolytic virotherapy promotes intratumoral T cell infiltration and improves anti-PD-1 immunotherapy. Cell (2017) 170(6):1109–19. doi: 10.1016/j.cell.2017.08.027 PMC8034392 28886381

[28] Parakrama R Fogel E Chandy C Augustine T Coffey M Tesfa L. Immune characterization of metastatic colorectal cancer patients post reovirus administration. BMC Cancer (2020) 20(1):569. doi: 10.1186/s12885-020-07038-2 32552875 PMC7301987

[29] Rascle P Woolley G Jost S Manickam C Reeves RK. NK cell education: Physiological and pathological influences. Front Immunol (2023) 14. doi: 10.3389/fimmu.2023.1087155 PMC9896005 36742337

[30] Osuna-Espinoza KY Rosas-Taraco AG. Metabolism of NK cells during viral infections. Front Immunol (2023) 14. doi: 10.3389/fimmu.2023.1064101 PMC9889541 36742317

[31] Tsukerman P Eisenstein EM Chavkin M Schmiedel D Wong E Werner M. Cytokine secretion and NK cell activity in human ADAM17 deficiency. Oncotarget (2015) 6(42):44151–60. doi: 10.18632/oncotarget.6629 PMC4792548 26683521

[32] Denaeghel S De Pelsmaeker S Van Waesberghe C Favoreel HW. Pseudorabies virus infection causes downregulation of ligands for the activating nk cell receptor nkg2d. Viruses (2021) 13(2):266. doi: 10.3390/v13020266 33572245 PMC7915010

[33] Siemaszko J Marzec-przyszlak A Bogunia-kubik K. Nkg2d natural killer cell receptor—a short description and potential clinical applications. Cells (2021) 10(6):1420. doi: 10.3390/cells10061420 34200375 PMC8229527

[34] Schmiedel D Mandelboim O. NKG2D ligands-critical targets for cancer immune escape and therapy. Front Immunol (2018) 9:2040. doi: 10.3389/fimmu.2018.02040 30254634 PMC6141707

[35] Smith DW Chakravorty A Hayes M Hammerschmidt W Sugden B. The epstein-barr virus oncogene EBNA1 suppresses natural killer cell responses and apoptosis early after infection of peripheral B cells. Microbiol (N Y). (2021) 12(6):e0224321. doi: 10.1128/mBio.02243-21 PMC8593684 34781735

[36] Seidel E Le VTK Bar-On Y Tsukerman P Enk J Yamin R. Dynamic co-evolution of host and pathogen: HCMV downregulates the prevalent allele MICA∗008 to escape elimination by NK cells. Cell Rep (2015) 10(6):968–82. doi: 10.1016/j.celrep.2015.01.029 PMC4641326 25683719

[37] Chaouat AE Seliger B Mandelboim O Schmiedel D. The HHV-6A proteins U20 and U21 target NKG2D ligands to escape immune recognition. Front Immunol (2021) 12. doi: 10.3389/fimmu.2021.714799 PMC8554080 34721381

[38] Diab M Schmiedel D Seidel E Bacharach E Mandelboim O. Human metapneumovirus escapes NK cell recognition through the downregulation of stress-induced ligands for NKG2D. Viruses (2020) 12(7):781. doi: 10.3390/v12070781 32698530 PMC7412239

[39] Dassa L Seidel E Oiknine-Djian E Yamin R Wolf DG Le-Trilling VTK. The human cytomegalovirus protein UL148A downregulates the NK cell-activating ligand MICA to avoid NK cell attack. J Virol (2018) 92(17):e00162–18. doi: 10.1128/JVI.00162-18 PMC6096798 29950412

[40] Bar-On Y Charpak-Amikam Y Glasner A Isaacson B Duev-Cohen A Tsukerman P. NKp46 recognizes the sigma1 protein of reovirus: implications for reovirus-based cancer therapy. J Virol (2017) 91(19):e01045–17. doi: 10.1128/JVI.01045-17 PMC5599737 28724773

[41] Long S Gu Y An Y Lin X Chen X Wang X. Reovirus enhances cytotoxicity of natural killer cells against colorectal cancer via TLR3 pathway. J Transl Med (2021) 19(1):185. doi: 10.1186/s12967-021-02853-y 33933132 PMC8088708

[42] Parrish C Scott GB Migneco G Scott KJ Steele LP Ilett E. Oncolytic reovirus enhances rituximab-mediated antibody-dependent cellular cytotoxicity against chronic lymphocytic leukaemia. Leukemia (2015) 29(9):1799–810. doi: 10.1038/leu.2015.88 PMC4490165 25814029

[43] Rajani K Parrish C Kottke T Thompson J Zaidi S Ilett L. Combination therapy with reovirus and Anti-PD-1 blockade controls tumor growth through innate and adaptive immune responses. Mol Ther (2016) 24(1):166–74. doi: 10.1038/mt.2015.156 PMC4754544 26310630

[44] Tomaz D Pereira PM Guerra N Dyson J Gould K Henriques R. Nanoscale colocalization of NK cell activating and inhibitory receptors controls signal integration. Front Immunol (2022) 13:868496. doi: 10.3389/fimmu.2022.868496 35720315 PMC9198454

[45] Kotzur R Duev-Cohen A Kol I Reches A Mandelboim O Stein N. NK-92 cells retain vitality and functionality when grown in standard cell culture conditions. PloS One (2022) 17(3):e0264897. doi: 10.1371/journal.pone.0264897 35294457 PMC8926178

[46] Berard A Coombs KM. MamMalian reoviruses: Propagation, quantification, and storage. Curr Protoc Microbiol (2009) SUPPL. 14):1–18. doi: 10.1002/9780471729259.mc15c01s14 19653214

[47] Mendoza EJ Manguiat K Wood H Drebot M. Two detailed plaque assay protocols for the quantification of infectious SARS-coV-2. Curr Protoc Microbiol (2020) 57(1):ecpmc105. doi: 10.1002/cpmc.105 32475066 PMC7300432

[48] Lankry D Rovis TL Jonjic S Mandelboim O. The interaction between CD300a and phosphatidylserine inhibits tumor cell killing by NK cells. Eur J Immunol (2013) 43(8):2151–61. doi: 10.1002/eji.201343433 PMC3809832 23640773

[49] Prestwich RJ Errington F Ilett EJ Morgan RSM Scott KJ Kottke T. Tumor infection by oncolytic reovirus primes adaptive antitumor immunity. Clin Cancer Res (2008) 14(22):7358–66. doi: 10.1158/1078-0432.CCR-08-0831 PMC2701231 19010851

[50] Samson A Scott KJ Taggart D West EJ Wilson E Nuovo GJ. Intravenous delivery of oncolytic reovirus to brain tumor patients immunologically primes for subsequent checkpoint blockade. Sci Transl Med (2018) 10(422):eaam7577. doi: 10.1126/scitranslmed.aam7577 29298869 PMC6276984

[51] Pearson JRD Regad T. Targeting cellular pathways in glioblastoma multiforme. Signal Transduction Targeted Ther (2017) 2:17040. doi: 10.1038/sigtrans.2017.40 PMC5661637 29263927

[52] Hah YS Cho HY Lim TY Park DH Kim HM Yoon J. Induction of melanogenesis by rapamycin in human MNT-1 melanoma cells. Ann Dermatol (2012) 24(2):151–7. doi: 10.5021/ad.2012.24.2.151 PMC3346904 22577264

[53] Sen Santara S Lee DJ Crespo Â Hu JJ Walker C Ma X. The NK cell receptor NKp46 recognizes ecto-calreticulin on ER-stressed cells. Nature (2023) 616:348–56. doi: 10.1038/s41586-023-05912-0 PMC10165876 37020026

[54] Goitea VE Hallak ME. Calreticulin and arginylated calreticulin have different susceptibilities to proteasomal degradation. J Biol Chem (2015) 290(26). doi: 10.1074/jbc.M114.626127 PMC4481237 25969538

[55] Schmechel S Chute M Skinner P Anderson R Schiff L. Preferential Translation of Reovirus mRNA by a sigma3-Dependent Mechanism. VIROLOGY (1997) 232(1):62–73. doi: 10.1006/viro.1997.8531 9185589

[56] Baugh R Khalique H Seymour LW. Convergent evolution by cancer and viruses in evading the nkg2d immune response. Cancers (2020) 12:1–27. doi: 10.3390/cancers12123827 PMC7766243 33352921

[57] Lee CH Raghunathan K Taylor GM French AJ Tenorio R De Castro IF. Reovirus nonstructural protein s ns recruits viral rna to replication organelles. mBio (2021) 12(4):e0140821. doi: 10.1128/mBio.01408-21 34225484 PMC8406312

[58] Zamora PF Hu L Knowlton JJ Lahr RM Moreno RA Berman AJ. Reovirus nonstructural protein NS acts as an RNA stability factor promoting viral genome replication. J Virol (2018) 92(15):e00563–18. doi: 10.1128/JVI.00563-18 PMC6052290 29769334

[59] Brentano L Noah DL Brown EG Sherry B. The reovirus protein 2, encoded by the M1 gene, is an RNA-binding protein. J OF VIROLO (1998) 72(10):8354–7. doi: 10.1128/JVI.72.10.8354-8357.1998 PMC110211 9733883

[60] Lemay G Danis C. Reovirus 21 protein: affinity for double-stranded nucleic acids by a small amino-terminal region of the protein independent from the zinc finger motif. J Gen Virol (1994) 75(Pt 11):3261–6. doi: 10.1099/0022-1317-75-11-326 7964637

[61] Broering TJ Kim J Miller CL Piggott CDS Dinoso JB Nibert ML. Reovirus nonstructural protein μNS recruits viral core surface proteins and entering core particles to factory-like inclusions. J Virol (2004) 78(4):1882–92. doi: 10.1128/JVI.78.4.1882-1892.2004 PMC369481 14747553

[62] Phillips MB Stuart JD Simon EJ Boehme KW. Nonstructural protein σ1s is required for optimal reovirus protein expression. J Virol (2018) 92(7):e02259–17. doi: 10.1128/JVI.02259-17 29321319 PMC5972882

[63] Luongo CL Reinisch KM Harrison SC Nibert ML. Identification of the guanylyltransferase region and active site in reovirus mRNA capping protein λ2. J Biol Chem (2000) 275(4):2804–10. doi: 10.1074/jbc.275.4.2804 10644745

[64] Kloess S Kretschmer A Stahl L Fricke S Koehl U. CAR-expressing natural killer cells for cancer retargeting. Transfusion Med Hemother (2019) 46:4–13. doi: 10.1159/000495771 PMC6558329 31244577

[65] Solocinski K Padget MR Fabian KP Wolfson B Cecchi F Hembrough T. Overcoming hypoxia-induced functional suppression of NK cells. J Immunother Cancer (2020) 8(1):e000246. doi: 10.1136/jitc-2019-000246 32345623 PMC7213912

[66] Fernández A Navarro-zapata A Escudero A Matamala N Ruz-caracuel B Mirones I. Optimizing the procedure to manufacture clinical-grade NK cells for adoptive immunotherapy. Cancers (Basel) (2021) 13(3):577. doi: 10.3390/cancers13030577 33540698 PMC7867223

[67] Nachmani D Gutschner T Reches A Diederichs S Mandelboim O. RNA-binding proteins regulate the expression of the immune activating ligand MICB. Nat Commun (2014) 5:4186. doi: 10.1038/ncomms5186 24924487 PMC4064450

[68] Stern-Ginossar N Gur C Biton M Horwitz E Elboim M Stanietsky N. Human microRNAs regulate stress-induced immune responses mediated by the receptor NKG2D. Nat Immunol (2008) 9(9):1065–73. doi: 10.1038/ni.1642 18677316

[69] Wensveen FM Jelenčić V Polić B. NKG2D: A master regulator of immune cell responsiveness. Front Immunol (2018) 9. doi: 10.3389/fimmu.2018.00441 PMC5852076 29568297

[70] Eagle RA Trowsdale J. Promiscuity and the single receptor: NKG2D. Nat Immunol (2007) 7:737–44. doi: 10.1038/nri2144 17673918

[71] Lauer UM Beil J. Oncolytic viruses: Challenges and considerations in an evolving clinical landscape. Future Oncol (2022) 18:2713–32. doi: 10.2217/fon-2022-0440 35818970

[72] Gujar SA Marcato P Pan D Lee PWK. Reovirus virotherapy overrides tumor antigen presentation evasion and promotes protective antitumor immunity. Mol Cancer Ther (2010) 9(11):2924–33. doi: 10.1158/1535-7163.MCT-10-0590 20978162

[73] Errington F Steele L Prestwich R Harrington KJ Pandha HS Vidal L. Reovirus activates human dendritic cells to promote innate antitumor immunity 1. J Immunol (2008) 180:6018–26. doi: 10.4049/jimmunol.180.9.6018 18424722

[74] Brigleb PH Kouame E Fiske KL Taylor GM Urbanek K Medina Sanchez L. NK cells contribute to reovirus-induced IFN responses and loss of tolerance to dietary antigen. JCI Insights (2022) 7(16):e159823. doi: 10.1172/jci.insight.159823 PMC9462493 35993365

[75] Wiedemann GM. Localization matters: epigenetic regulation of natural killer cells in different tissue microenvironments. Front Immunol (2022) 13:913054. doi: 10.3389/fimmu.2022.913054 35707540 PMC9191276

[76] Bourayou E Golub R. Inflammatory-driven NK cell maturation and its impact on pathology. Front Immunol (2022) 13:1061959. doi: 10.3389/fimmu.2022.1061959 36569860 PMC9780665

[77] Le T Reeves RK McKinnon LR. The functional diversity of tissue-resident natural killer cells against infection. Immunology (2022) 167:28–39. doi: 10.1111/imm.13523 35751452

[78] Pinto C Estrada MF Brito C. *In vitro* and ex vivo models – the tumor microenvironment in a flask. Adv Exp Med Biol (2020) 1219:431–43. doi: 10.1007/978-3-030-34025-4_23 32130713

[79] Ohashi M Eagle RA Trowsdale J. Post-translational modification of the NKG2D ligand RAET1G leads to cell surface expression of a glycosylphosphatidylinositol-linked isoform. J Biol Chem (2010) 285(22):16408–15. doi: 10.1074/jbc.M109.077636 PMC2878053 20304922

[80] Giannattasio A Weil S Kloess S Ansari N Stelzer EHK Cerwenka A. Cytotoxicity and infiltration of human NK cells in *in vivo*-like tumor spheroids. BMC Cancer (2015) 15:351. doi: 10.1186/s12885-015-1321-y 25933805 PMC4422268

[81] Fielding CA Weekes MP Nobre LV Ruckova E Wikie GS Paulo JA. Control of immune ligands by members of a cytomegalovirus gene expansion suppresses natural killer cell activation. Elife (2017) 6:e22206. doi: 10.7554/eLife.22206 28186488 PMC5367895

[82] Boehme KW Ikizler M Kobayashi T Dermody TS. Reverse genetics for mamMalian reovirus. Methods Acad Press Inc (2011) 55(2):109–13. doi: 10.1016/j.ymeth.2011.07.002 PMC3208765 21798351

