# Supplementary material for: Reovirus infection of tumor cells reduces the expression of NKG2D ligands, leading to impaired NK-cell cytotoxicity and functionality
Source: Front Immunol. 2023 Sep 11;14:1231782. doi: 10.3389/fimmu.2023.1231782 (PMC10518469; doi:10.3389/fimmu.2023.1231782)
Supplement: Supplementary file 1 [file Presentation_1.pdf]

Figure S1.

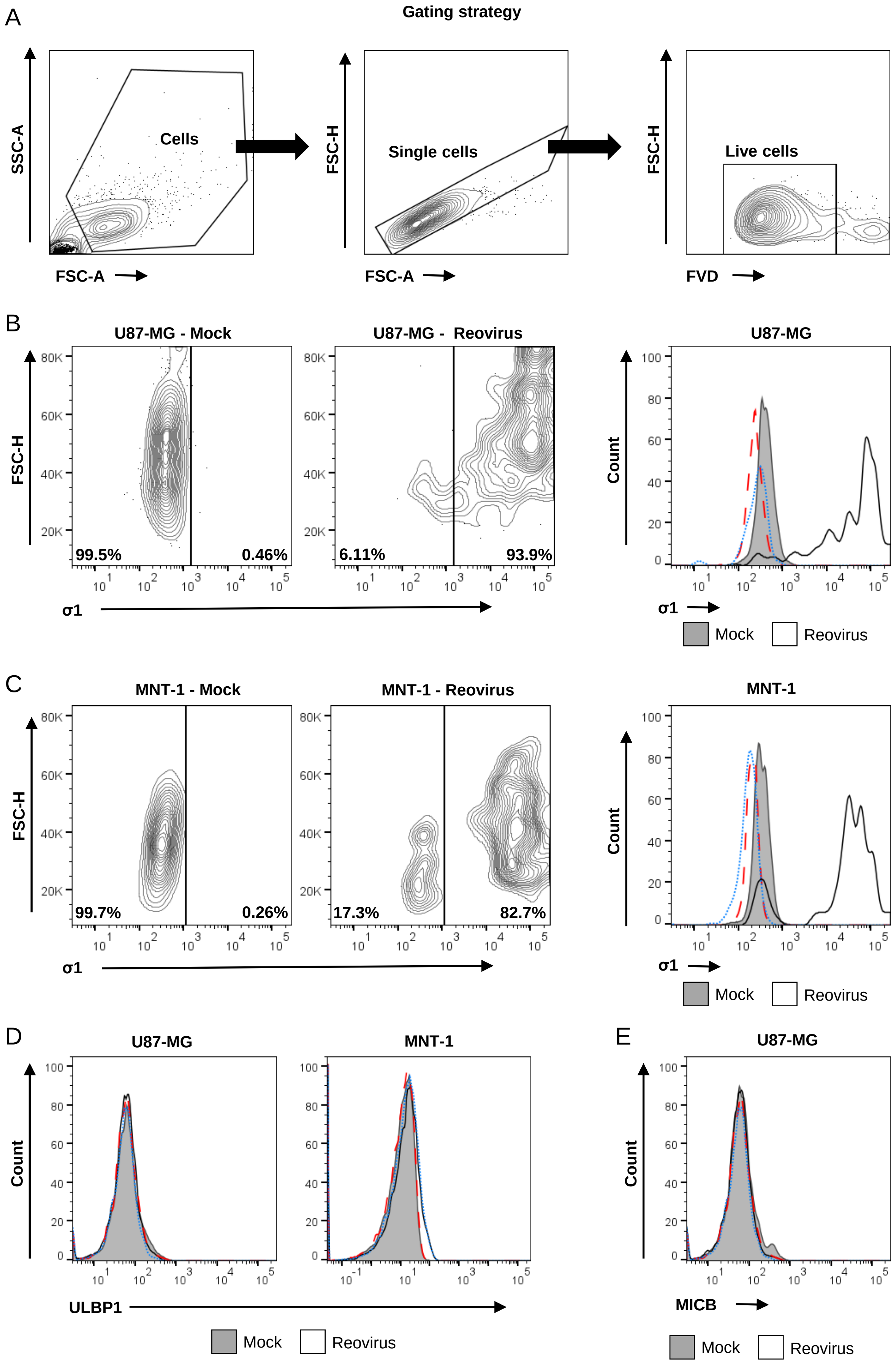
A
Gating strategy
SSC-A
FSC-A
Cells
FSC-H
FSC-A
Single cells
FSC-H
FVD
Live cells
B
U87-MG - Mock
U87-MG - Reovirus
U87-MG
FSC-H
99.5%
0.46%
6.11%
93.9%
σ1
Count
Mock
Reovirus
C
MNT-1 - Mock
MNT-1 - Reovirus
MNT-1
99.7%
0.26%
17.3%
82.7%
D
U87-MG
MNT-1
ULBP1
E
U87-MG
MICB

Figure S2.

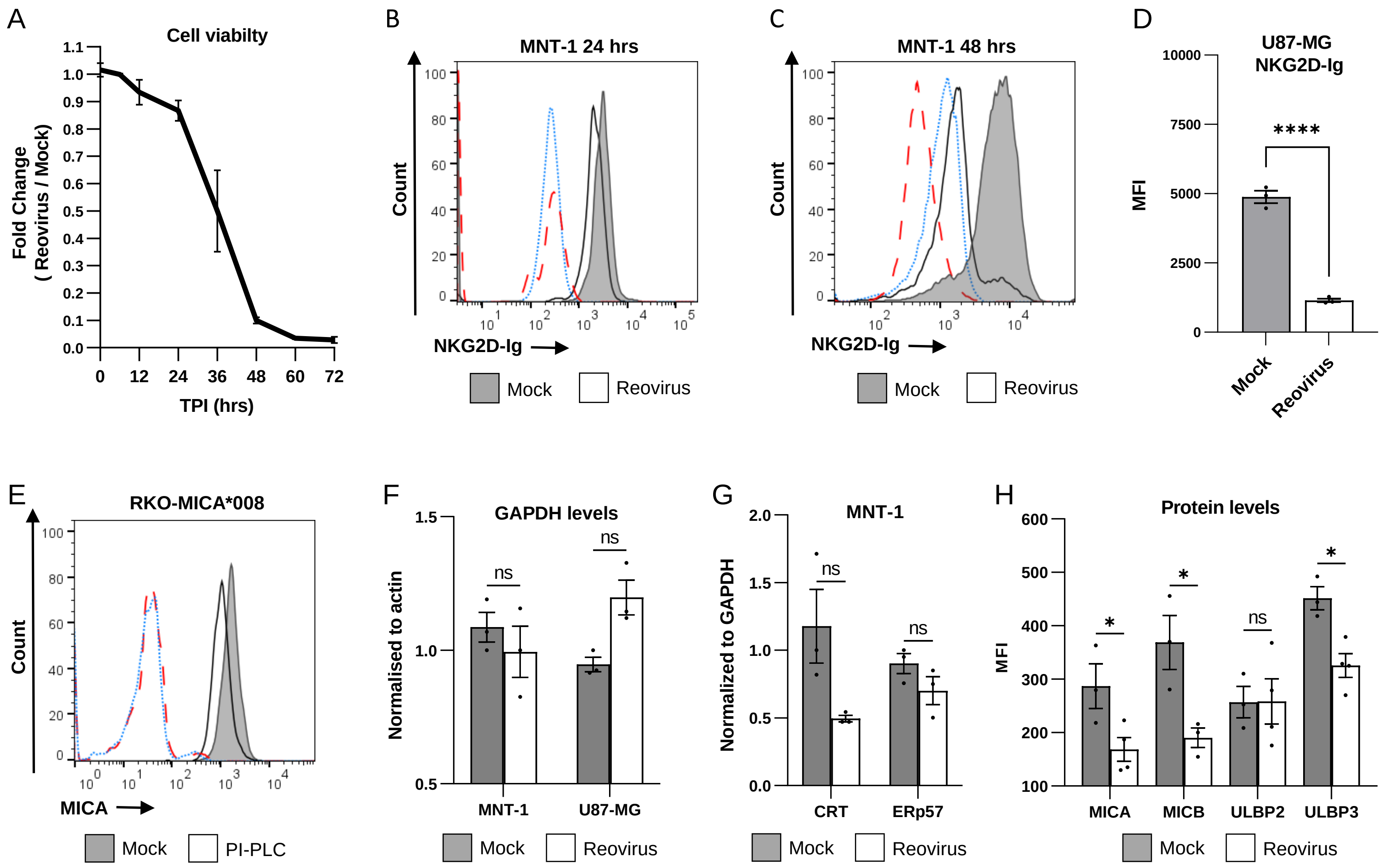

A
Cell viabilty
Fold Change (Reovirus / Mock)
TPI (hrs)
B
MNT-1 24 hrs
Count
NKG2D-Ig
Mock
Reovirus
C
MNT-1 48 hrs
Count
NKG2D-Ig
Mock
Reovirus
D
U87-MG NKG2D-Ig
MFI
****
Mock
Reovirus
E
RKO-MICA*008
Count
MICA
Mock
PI-PLC
F
GAPDH levels
Normalised to actin
ns
ns
MNT-1
U87-MG
Mock
Reovirus
G
MNT-1
Normalized to GAPDH
ns
ns
CRT
ERp57
Mock
Reovirus
H
Protein levels
MFI
*
*
ns
*
MICA
MICB
ULBP2
ULBP3
Mock
Reovirus

Figure S3.

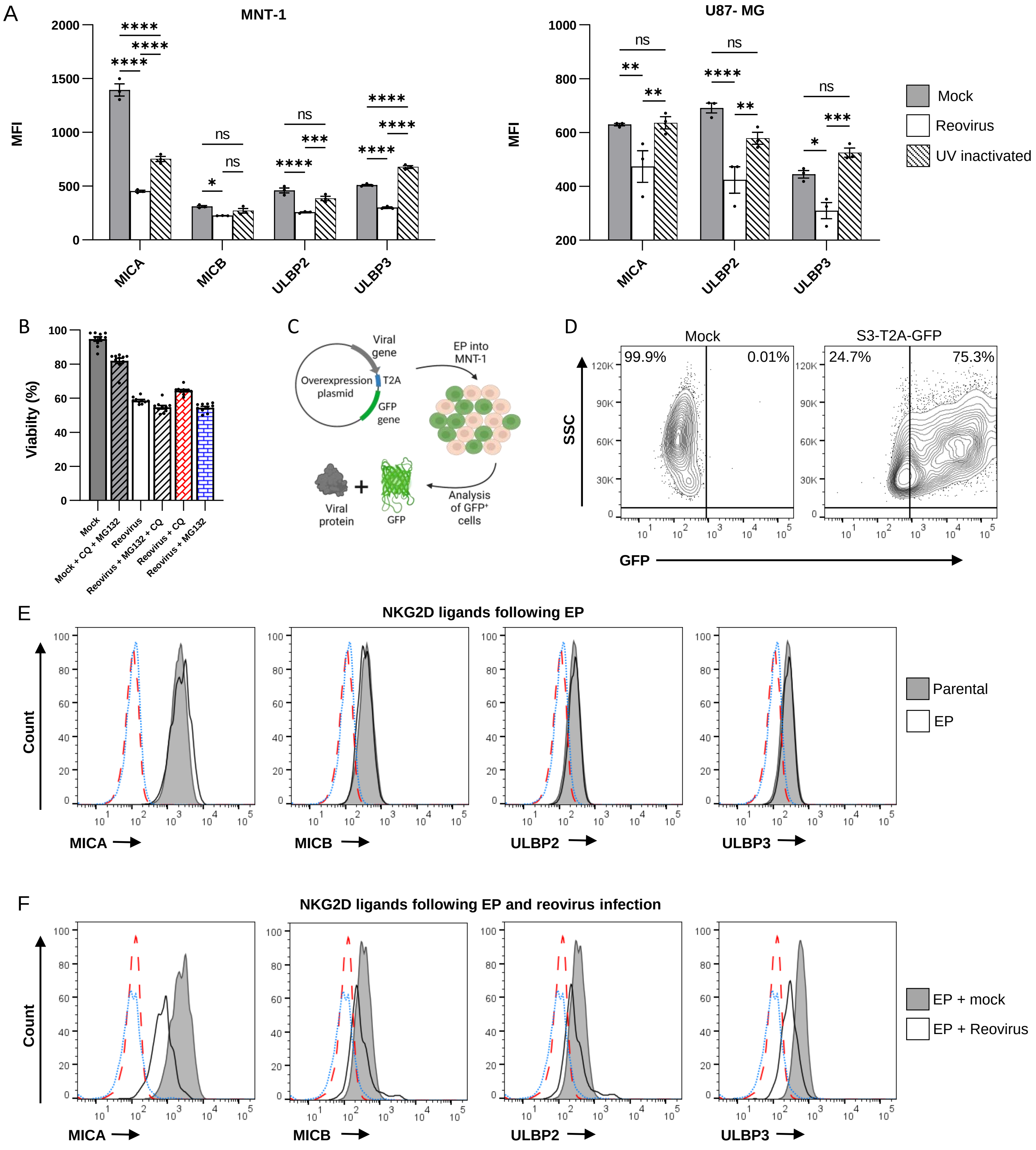

A
MNT-1
MFI
MICA
MICB
ULBP2
ULBP3
U87- MG
Mock
Reovirus
UV inactivated
B
Viability (%)
Mock
Mock + CQ + MG132
Reovirus
Reovirus + MG132 + CQ
Reovirus + CQ
Reovirus + MG132
C
Viral gene
Overexpression plasmid
T2A
GFP gene
EP into MNT-1
Viral protein
GFP
Analysis of GFP+ cells
D
Mock
S3-T2A-GFP
99.9%
0.01%
24.7%
75.3%
SSC
GFP
E
NKG2D ligands following EP
Count
Parental
EP
F
NKG2D ligands following EP and reovirus infection
EP + mock
EP + Reovirus

Figure S4.

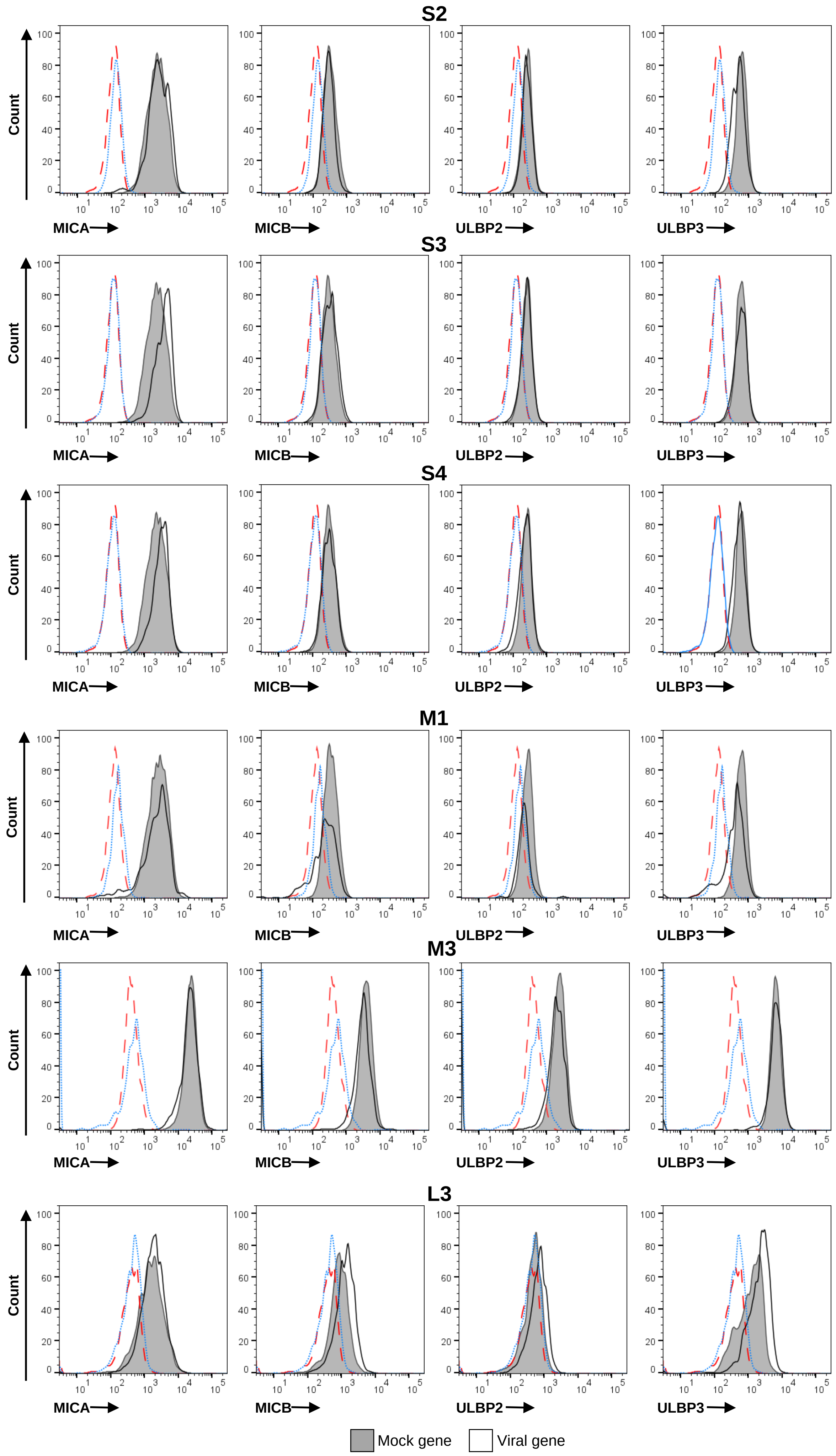
S2
S3
S4
M1
M3
L3
Count
MICA
MICB
ULBP2
ULBP3
Mock gene
Viral gene

Figure S4. Continued

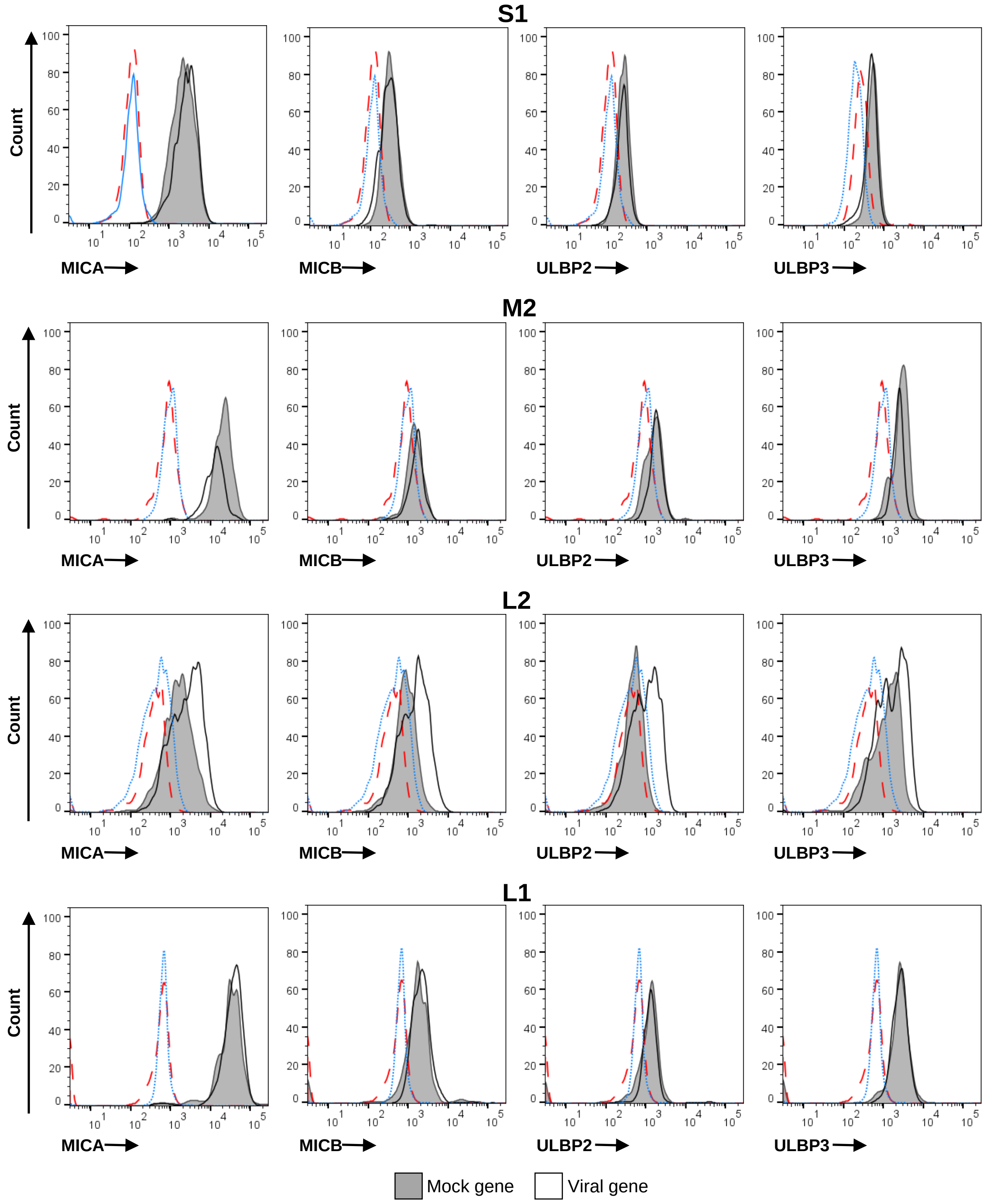

Figure S5.

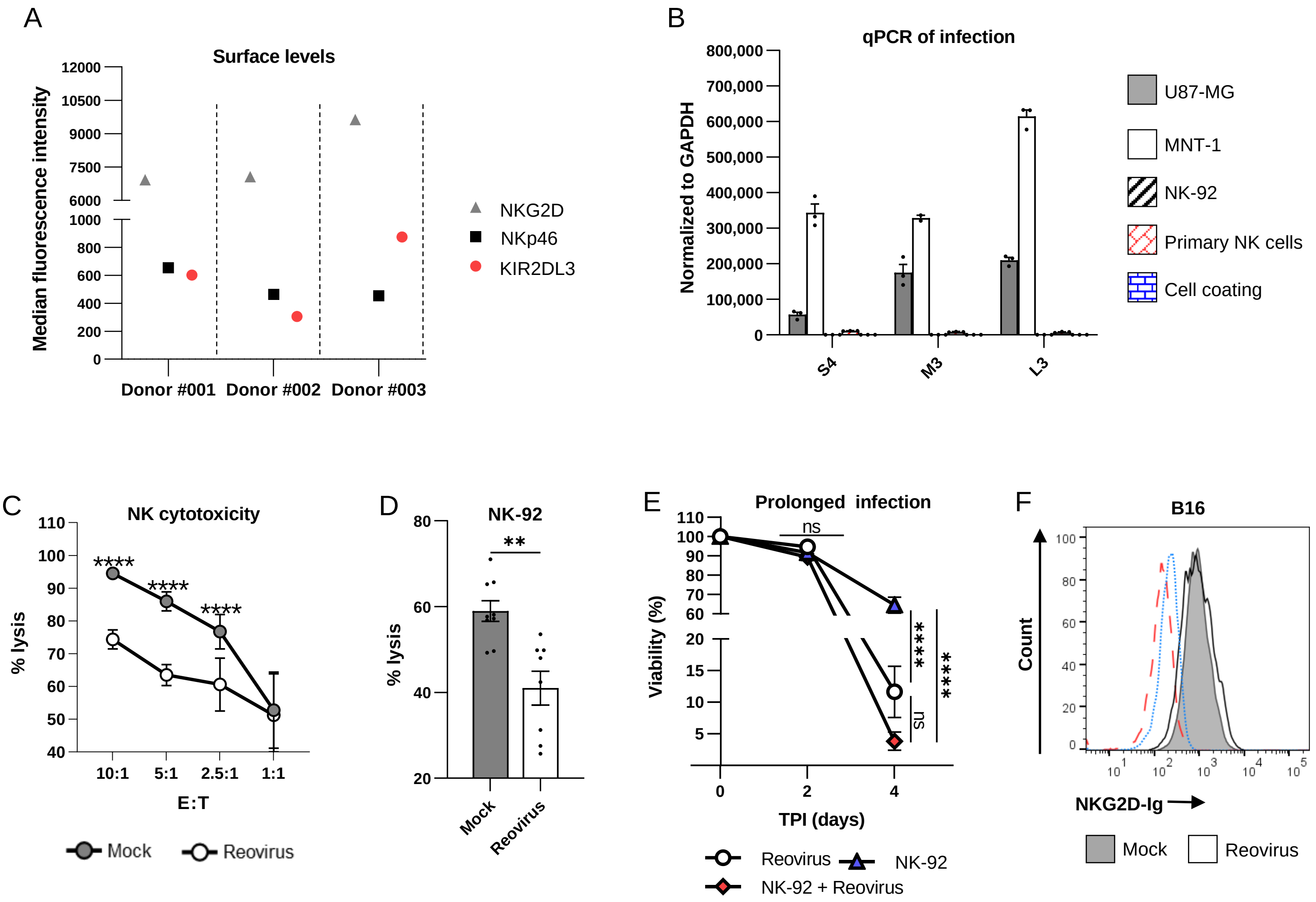
A
Surface levels
Median fluorescence intensity
Donor #001
Donor #002
Donor #003
NKG2D
NKp46
KIR2DL3
B
qPCR of infection
Normalized to GAPDH
S4
M3
L3
U87-MG
MNT-1
NK-92
Primary NK cells
Cell coating
C
NK cytotoxicity
% lysis
E:T
10:1
5:1
2.5:1
1:1
Mock
Reovirus
D
NK-92
% lysis
Mock
Reovirus
E
Prolonged infection
Viability (%)
TPI (days)
ns
Reovirus
NK-92
NK-92 + Reovirus
F
B16
Count
NKG2D-Ig
Mock
Reovirus

Figure S1.

(A) Gating strategy used in this study. Cells were selected using SSC-A and FSC-A, then further selected to single cells using FSC-H and FSC-A, followed by gating for live single cells which are negative for the FVD. Live single cells were further studied and analyzed. This gating strategy was applied in all experiments, followed by further selection if required. **(B)** Intra-cellular FACS staining for the σ1 protein of the reovirus in U87-MG cells, 48 hrs post infection with mock virus, or with reovirus**.** Grey histogram depicts mock-infected cells, black line depicts reovirus infected cells, red dashed-line depicts the background staining of mock-infected cells, blue dotted-line depicts the background staining of reovirus-infected cells. MOI=100 was used in these experiments. **(C)** Intra-cellular FACS staining for the σ1 protein of the reovirus in MNT-1 cells, 48 hrs post infection with mock virus, or with reovirus. Grey histogram depicts mock-infected cells, black line depicts reovirus infected cells, red dashed-line depicts the background staining of mock-infected cells, blue dotted-line depicts the background staining of reovirus-infected cells. MOI=100 was used in these experiments. **(D)** FACS staining for MICB in U87-MG cells 48 hrs post infection. Grey histogram depicts mock-infected cells, black line depicts reovirus infected cells, red dashed-line depicts the background staining of mock-infected cells, blue dotted-line depicts the background staining of reovirus-infected cells. Shown is 1 representative staining out of 3 that were performed. MOI=100 was used in these experiments.**(E)** FACS staining for ULBP-1 in U87-MG cells and in MNT-1 cells 48 hrs post infection. Grey histogram depicts mock-infected cells, black line depicts reovirus infected cells, red dashed-line depicts the background staining of mock-infected cells, blue dotted-line depicts the background staining of reovirus-infected cells. Shown is 1 representative staining out of 3 that were performed. MOI=100 was used in these experiments.

Figure S2.

(A) The kinetics of cell lysis of MNT-1 cells post reovirus-infection compared to mock-infected cells. The y axis shows fold change of the viable population in both samples, calculated as the percentage of viable reovirus-infected cells divided by MFI of mock-infected cells. The x axis shows time post infection (TPI) in hrs. Shown are 3 pooled experiments in each time point. MOI=200 was used in these experiments. **(B)** FACS staining with human NKG2D-Ig fusion protein after incubation with mock-infected and reovirus-infected MNT-1 cells 24 hrs post infection. Grey histogram depicts mock-infected cells, black line depicts reovirus infected cells, red dashed-line depicts the background staining of mock-infected cells, blue dotted-line depicts the background staining of reovirus-infected cells. Shown is 1 representative staining out of 3 that were performed. MOI=100 was used in these experiments. **(C)** FACS staining with human NKG2D-Ig fusion protein after incubation with mock-infected and reovirus-infected MNT-1 cells, 48 hrs post infection. Grey histogram depicts mock-infected cells, black line depicts reovirus infected cells, red dashed-line depicts the background staining of mock-infected cells, blue dotted-line depicts the background staining of reovirus-infected cells. Shown is 1 representative staining out of 3 that were performed. MOI=200 was used in these experiments. **(D)** Summary of the MFI of staining of with NKG2D-Ig fusion protein after incubation with mock-infected and reovirus-infected U87-MG cells, 48 hrs post infection. The grey columns depict mock-infected cells and the white columns depicts reovirus-infected cells. Shown is a summary of 3 experiments. MOI=200 was used in these experiments. One-tailed unpaired Student's t test was used to determine statistical significance. *p<0.05, **p<0.01, ***p<0.005 ****p<0.0001. Each dot on the plot depicts the result of an independent experiment. Data is presented as mean ± SEM. **(E)** FACS staining of MICA in RKO*008 cells post a 4 hrs-treatment with PI-PLC. Grey histogram depicts mock-treated cells, black line depicts PI-PLC treated cells, red dashed-line depicts the background staining of mock-treated cells, blue dotted-line depicts the background staining of PI-PLC treated cells. **(F)** Analysis of

qPCR of the GAPDH normalized to actin levels at 48 hrs post infection. The grey columns depict mock-infected cells, the white columns depict reovirus-infected cells. Shown is a summary of 3 experiments. MOI=100 was used in these experiments. Mann-Whitney tests were used to determine statistical significance. *p<0.05, **p<0.01, ***p<0.005, ****p<0.0001. Each dot on the plot depicts the result of an independent experiment. Data is presented as mean ± SEM. **(G)** Analysis of qPCR of CRT and ERp57 normalized to GAPDH at 48 hrs post infection. The grey columns depict mock-infected cells, the white columns depict reovirus-infected cells. Shown is a summary of 3 experiments MOI=100 was used in these experiments. Mann-Whitney tests were used to determine statistical significance. *p<0.05, **p<0.01, ***p<0.005, ****p<0.0001. Each dot on the plot depicts the result of an independent experiment. Data is presented as mean ± SEM. **(H)** Protein levels of NKG2D-ligands (MICA, MICB, ULBP2, ULBP3) in mock-infected or reovirus-infected MNT-1 cells, 48 hrs post infection. Grey columns depict mock-infected cells, columns depict reovirus-infected cells. The y axis depicts the MFI. The x axis depicts NKG2D-ligands. Shown is pooled data from 4 independent experiments. Two-tailed unpaired Student's t test was used to determine statistical significance. *p<0.05, **p<0.01, ***p<0.005, ****p<0.0001. Each dot on the plot depicts the result of an independent experiment. Data is presented as mean ± SEM.

Figure S3.

(A) Summary of the MFI of staining of NKG2D ligand in MNT-1 cells and in U87-MG cells, 48 hrs post infection with reovirus, with UV-inactivated reovirus or with mock. Grey columns depict mock-infected cells, white columns depict reovirus-infected cells, patterned columns depict cells infected with UV inactivated virus. Shown is a summary of 3 experiments. MOI=100 was used in these experiments. Two-way ANOVA followed by Tukey's test were

used to determine statistical significance. *$p<0.05$, **$p<0.01$, ***$p<0.005$, ****$p<0.0001$. Each dot on the plot depicts the result of an independent experiment. Data is presented as mean ± SEM. **(B)** Summary the viability percentage of reovirus-infected and mock-infected cells exposed to 100 µM CQ, 10 µM MG132, or a combination of both treatments. The y axis depicts percentage of the viable population from the parent population. The various treatment groups are depicted in the x axis. Each dot on the plot depicts the result of an independent experiment. **(C)** Schematic illustration of the method used to study the effect of separate viral genes on MNT-1 cells. **(D)** Representative FACS stain for GFP^+ cells, 72 hrs post electroporation EP. of mock plasmid, or S3-2A-GFP plasmid into MNT-1 cells. **(E)** FACS staining for NKG2D ligands in MNT-1 cells at 72 hrs post EP. Grey histogram depicts parental cells, black line depicts cells following EP, red dashed-line depicts the background staining of parental cells, blue dotted-line depicts the background staining of cells following EP. Shown is 1 representative staining out of 3 that were performed. **(F)** FACS staining for NKG2D ligands in MNT-1 cells following EP and reovirus infection. Grey histogram depicts cells 72 hrs post EP, black line depicts cells 72 hrs EP and 48 hrs post rovirus infection, red dashed-line depicts the background staining of cells following EP, blue dotted-line depicts the background staining of cells following EP and reovirus infection. Shown is 1 representative staining out of 3 that were performed. MOI=100 was used in these experiments.

Figure S4.

FACS staining for NKG2D ligands in MNT-1 cells 72 hrs EP with vectors coding for reovirus's genes. Grey histogram depicts GFP^+ cells following EP with a mock-T2A-GFP plasmid, black line depicts GFP^+ cells following EP with vectors coding the viral gene-T2A-GFP, red dashed-line depicts the background staining of cells following EP with a mock-T2A-GFP plasmid, blue dotted-line depicts the background staining of cells EP with viral gene-T2A-GFP plasmid. Shown is 1 representative staining out of 3 that were performed.

Figure S5.

(A) Summary of surface levels of NK receptors of NK cells derived from Donors #001, #002 and #003. The y axis depicts the median fluorescence intensity of FACS staining against NKG2D, NKp46 and KIR2DL3. The x axis depicts the donors. Grey triangle depicts NKG2D. Black square depicts NKp46. Red circle depicts KIR2DL3. Shown is summary of 3 experiments that were performed. **(B)** Analysis of qPCR of the viral genes S4, M3 and L3, normalized to GAPDH levels at 48 hrs post infection. The y axis depicts the fold change of expression levels in infected cells, divided by expression levels in cells coated with the reovirus on ice for 1 hr. The x axis depicts the different cell types used in this experiment. Shown is a summary of 3 experiments. MOI=100 was used in these experiments. Each dot on the plot depicts the result of an independent experiment. Data is presented as mean ± SEM. **(C)** Pooling of data acquired from NK cytotoxicity assay for mock-infected or reovirus-infected MNT-1 cells 4 hrs after coculturing with NK cells derived from 3 different donors (#001, #002, #003), at different effector to target (E:T) ratios that are shown in the x-axis. The y axis depicts cell lysis attributed to NK-cytotoxicity. Grey dots depict mock-infected cells, white dots depict reovirus-infected cells. Shown is 1 out of 3 experiments that were performed, each consisting of 3 biological triplicates, with cells derived from the 3 different donors. MOI=100 was used in these experiments. Two-way ANOVA followed by Sidak's test were used to determine statistical significance. $^{*}p<0.05$, $^{**}p<0.01$, $^{***}p<0.005$, $^{****}p<0.0001$. Each dot on the plot depicts the mean. Data is presented as mean ± SEM. **(D)** NK cytotoxicity assay for mock-infected or reovirus-infected MNT-1 cells 4 hrs after coculturing with NK-92 cells at the E:T ratio of 2.5:1. MOI=100 was used in these experiments. Two-tailed unpaired Student's t test was used to determine statistical significance. $^{*}p<0.05$, $^{**}p<0.01$, $^{***}p<0.005$, $^{****}p<0.0001$. Each dot on the plot depicts the result of an independent experiment. Data is presented as mean ± SEM. **(E)** Summary the viability percentage of reovirus-infected and mock-infected cells

following prolonged exposure to NK-92 cells, reovirus or a combination of NK-92 and reovirus. The y axis depicts percentage of the viable population from the parent population. The x axis depicts the time post infection (TPI) in days. MOI=1 was used in these experiments. MOI=0.5 was used in these experiments. Two-way ANOVA followed by Tukey's test were used to determine statistical significance. *p<0.05, **p<0.01, ***p<0.005, ****p<0.0001. Data is presented as mean ± SEM. **(F)** FACS staining with murine NKG2D-Ig fusion protein after incubation with mock-infected and reovirus-infected B16 cells, 48 hrs post infection. Grey histogram depicts mock-infected cells, black line depicts reovirus infected cells, red dashed-line depicts the background staining of mock-infected cells, blue dotted-line depicts the background staining of reovirus-infected cells. Shown is 1 representative staining out of 3 that were performed. MOI=100 was used in these experiments.
